# Gγ recruitment systems specifically select PPI and affinity-enhanced candidate proteins that interact with membrane protein targets

**DOI:** 10.1038/srep16723

**Published:** 2015-11-19

**Authors:** Misato Kaishima, Jun Ishii, Nobuo Fukuda, Akihiko Kondo

**Affiliations:** 1 Department of Chemical Science and Engineering, Graduate School of Engineering, Kobe University, 1-1 Rokkodai, Nada, Kobe, Japan; 2 Organization of Advanced Science and Technology, Kobe University, 1-1 Rokkodai, Nada, Kobe, Japan; 3 Biomedical Research Institute, National Institute of Advanced Industrial Science and Technology (AIST), Higashi, Tsukuba, Japan

## Abstract

Protein-protein interactions (PPIs) are crucial for the vast majority of biological processes. We previously constructed a Gγ recruitment system to screen PPI candidate proteins and desirable affinity-altered (affinity-enhanced and affinity-attenuated) protein variants. The methods utilized a target protein fused to a mutated G-protein γ subunit (Gγ_cyto_) lacking the ability to localize to the inner leaflet of the plasma membrane. However, the previous systems were adapted to use only soluble cytosolic proteins as targets. Recently, membrane proteins have been found to form the principal nodes of signaling involved in diseases and have attracted a great deal of interest as primary drug targets. Here, we describe new protocols for the Gγ recruitment systems that are specifically designed to use membrane proteins as targets to overcome previous limitations. These systems represent an attractive approach to exploring novel interacting candidates and affinity-altered protein variants and their interactions with proteins on the inner side of the plasma membrane, with high specificity and selectivity.

Protein-protein interactions (PPIs) are attracting increased attention in drug discovery studies. PPIs have functions in the regulation of cellular states involved in various diseases^1^,^2^. In particular, membrane-mediated PPIs play central roles in vital biological processes and are prime drug targets. For example, tumorigenesis is often the result of gene mutations that lead to alterations in membrane PPIs and aberrant signaling cascades^3^. Because the molecules that control (inhibit or activate) these membrane PPIs can be used as drug candidates, rapid and unbiased screening of these molecules is essential for drug development.

The major targets of membrane proteins are G-protein-coupled receptors (GPCRs), ion channels, transporters, receptor serine/threonine and tyrosine protein kinases^4^,^5^ (e.g. epidermal growth factor receptor (EGFR)^6^,^7^, human epidermal growth factor receptor 2 (HER2)^8^,^9^, and vascular endothelial growth factor receptor (VEGFR)^10^,^11^). The extracellular domains of these transmembrane proteins are commonly targeted to identify agonistic and antagonistic ligands. However, recently developed drug therapies have increasingly targeted the intracellular domains (kinase domains) of these transmembrane proteins to control interactions with the components of downstream signaling cascades^12^. Similarly, membrane-associated proteins, such as guanine nucleotide-binding protein (G-protein), small GTPases, kinase proteins and other signal transducers, hold enormous potential for use in the development of novel drugs. As a representative example, protein kinases are responsible for the reversible phosphorylation of proteins via PPIs and have a strong relationship with growth, infiltration and apoptosis in cancer cells. A multitude of these membrane-associated proteins are involved in various diseases and are often associated with the inner side of the plasma membrane^13^. Several kinase and GTPase inhibitors have been developed in the pharmaceutical industry^14^,^15^,^16^. More recently, intracellular antibodies (intrabodies), which can inhibit signal transducers, including membrane-associated proteins, have been studied as valuable tools for controlling PPIs inside cells^17^,^18^,^19^. Thus, molecules that can control the PPIs of transmembrane and membrane-associated proteins on the inner side of the plasma membrane have a potential to become an important group of drug targets.

Various useful screening systems for PPIs exist and have yielded significant findings^20^,^21^,^22^,^23^. These techniques are required for screening of large numbers of proteins and are preferable in the *in vivo* cellular context. In particular, yeast two-hybrid systems are the typical tools for such screening of candidate proteins *in vivo*^24^,^25^,^26^,^27^. Among them, split-ubiquitin system is a well-established, useful technique to screen the candidate proteins with the PPIs for membrane target proteins^28^,^29^. As in other yeast systems, small G-protein-based methods, including the Sos recruitment system and the Ras recruitment system, are occasionally used to study the PPIs of membrane proteins^23^,^30^,^31^. These methods remain useful alternatives to the original two-hybrid system; however, they suffer from technical complexities, such as the different temperatures required for growth and screening (25 °C and 36 °C), slow growth at suboptimal temperatures, obligatory replica-plating steps (glucose to galactose medium), and the total time required for the procedure (~7 days including precultivation)^32^,^33^,^34^. In addition to bioluminescence resonance energy transfer (BRET) and fluorescence resonance energy transfer (FRET)^35^, protein fragment complementation assays using split-GFP and split-luciferase^36^,^37^,^38^,^39^,^40^ are useful tools for detecting the association of two proteins in living cells and have the potential to resolve these limitations. Among the varied systems used, growth reporters are generally applicable to library screening because of their convenience. Our previously developed screening method using yeast heterotrimeric G-proteins, called the Gγ recruitment system^41^,^42^,^43^, also makes it possible to screen PPIs between a target protein and candidate proteins by the mating growth assay without false-positive clones. The details of the mechanism utilized for detecting PPIs are presented below.

The Gγ recruitment system for detecting PPIs is based on the fundamental principle that yeast pheromone (mating) signaling requires the localization of a complex consisting of the β- and γ-subunits of heterotrimeric G-proteins (Gβ/Gγ) to the inner leaflet of the plasma membrane^42^. In yeast, the G-protein-coupled receptor (GPCR) undergoes a conformational change after binding ligands and then activates heterotrimeric G-proteins. The activated G-proteins trigger the dissociation of the Gβ/Gγ complex from Gα concurrently with the exchange of GDP/GTP on the Gα subunit. The Gβ subunit (complexed with membrane-associated Gγ) then acts upon the effectors, thereby activating the downstream signaling cascade for mating^44^. Notably, localization of the Gβ/Gγ complex to the inner leaflet of the plasma membrane via the lipidation motif of the Gγ subunit is required for initiating G-protein signaling. Our Gγ recruitment system specifically makes use of a cytosolic truncated variant of Gγ (named Gγ_cyto_) that is fused to a soluble target protein of interest, ‘X’ (Gγ_cyto_-X), as shown in Fig. 1A. For the library, the candidate proteins (Y_1_) should be attached to the artificial lipidation site to ensure localization to the membrane (Fig. 1A). When an interaction occurs between target ‘X’ and candidate ‘Y_1_’, the Gγ_cyto_-X fusion protein brings Gβ to the membrane and induces subsequent activation of the pheromone signaling pathway. The promoted signaling can be detected by a fluorescent reporter assay or a mating growth assay after growth in simple glucose media at the optimal temperature (30 °C). Briefly, the expression of *GFP* under the control of a pheromone-responsive *FIG1* promoter or mating with intact haploid cells of the opposite mating type permits the detection of PPIs (Fig. 1A and Fig. S1). Because the localization of Gγ_cyto_ in the cytosol completely prevents this signaling activation, the Gγ recruitment system allows for extremely reliable, low-background growth screening that excludes false-positive candidates at the optimal temperature (30 °C)^42^. The procedures for screening involve simply mixing the different mating-type cells (recombinant **a**-cells and intact α-cells) and plating on selective media (~4 days including precultivation) (Fig. S1; **right**). The advanced system (competitor-introduced Gγ recruitment system), which additionally expresses an interaction competitor protein (Y_2_) in the cytosol (Fig. 2A), can offer highly selective screening for protein variants whose affinities have been intentionally altered to exceed the set threshold^41^. This approach is applicable to selectively screening affinity-enhanced or affinity-attenuated protein variants by exchanging the positions of the competitor protein and the library proteins (Y_1_ and Y_2_)^41^,^45^.

The localization of Gγ is of key importance for the low background of the Gγ recruitment system^42^. The previous Gγ recruitment system was limited to using only soluble cytosolic proteins as the target (X), as candidate proteins (Y_1_) should be expressed on the membrane (Fig. 1A). The competitor-introduced system also had a similar problem, restricting the target X to cytosolic proteins (Fig. 2A). Thus, these previous systems could not target membrane proteins. In the current study, we have reevaluated the Gγ recruitment system by changing the localization of target proteins from the cytosol to the membrane; however, the prior protocol did not work well. With the aim of expanding the applicability of the system, we considered new protocols for the Gγ recruitment systems that might be suitable for evaluating membrane proteins as targets. The updated method allows the Gγ recruitment system to be used in the analysis of both cytoplasmic and membrane target proteins.

## Results

### Selection of candidate proteins interacting with membrane protein targets using a previously established PPI-detecting Gγ recruitment system

**Figure 1:**
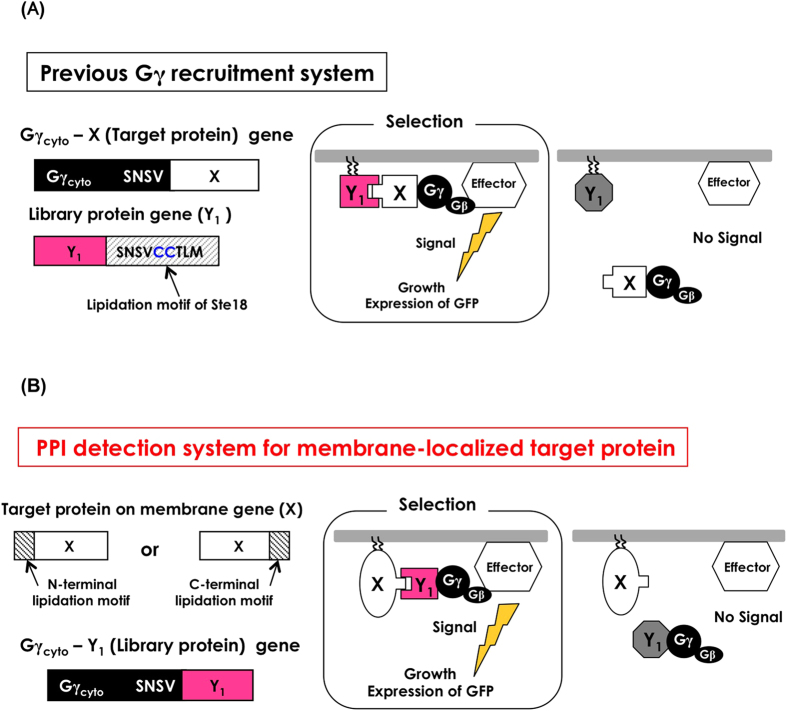
Schematic diagram of Gγ recruitment systems to detect PPIs of cytosolic or membrane target proteins. (**A**) Schematic outline of the previously established Gγ recruitment system for cytosolic target proteins. When target protein ‘X’ fused to Gγ_cyto_ interacts with candidate protein ‘Y_1_’, the Gβ and Gγ_cyto_ complex (Gβγ_cyto_) migrates to the inner leaflet of the plasma membrane and restores the signaling function. If protein ‘X’ cannot interact with protein ‘Y_1_’, Gβγ_cyto_ is released into the cytosol, and signaling is blocked. (**B**) Schematic outline of the Gγ recruitment system for membrane protein targets. When membrane target protein ‘X’ interacts with candidate protein ‘Y_1_’ fused to Gγ_cyto_, the Gβ and Gγ_cyto_ complex (Gβγ_cyto_) migrates to the inner leaflet of the plasma membrane and restores the signaling function. If membrane protein ‘X’ cannot interact with protein ‘Y_1_’, Gβγ_cyto_ is released into the cytosol, and signaling is blocked.

First, we tested whether the previous Gγ recruitment system could target membrane proteins. In the previous system, the Fc protein of human immunoglobulin G (IgG) and the Z domain of *Staphylococcus aureus* protein A (Z_WT_)^46^ were used for the PPI models. Several Z variants (Z_WT_, Z_K35A_, Z_I31A_ and Z_955_) with varied affinities for the Fc protein were also used for the PPI models (Z_WT_, 5.9 × 10^7^ M^−1^; Z_K35A_, 4.6 × 10^6^ M^−1^; Z_I31A_, 8.0 × 10^3^ M^−1^; and Z_955_, none)^47^,^48^. In contrast to the previous system, target protein ‘X’ was set to localize to the inner leaflet of the plasma membrane (previously, target ‘X’ was fused to Gγ_cyto_ in the cytosol), and candidate protein ‘Y_1_’ was fused to Gγ_cyto_ (previously, candidate protein ‘Y_1_’ was artificially localized to the inner leaflet of the membrane) (Fig. 1A,B). As the fictive model of target protein ‘X,’ the Fc fragment was fused to the lipidation motifs in this study (Fig. 1B). It was also notable that the lipidation motifs were fused to the Fc fragment at both the N-terminus (Gpa1p motif; Gpa1N) and the C-terminus (Ste18p motif; Ste18C) to test the accessibility between the Fc fragment and the Z variants (the C-terminal Ste18p motif was used to express the Z variants as the candidate ‘Y_1_’ proteins described in the previous study) (Fig. 1B). As the models of ‘Y_1_’ proteins for the candidate library, the Z variants were fused to the C-terminus of Gγ_cyto_ to express Gγ_cyto_-Y_1_ fusion proteins in the cytosol (Fig. 1B).

To express the target membrane proteins, the genes encoding the Fc fragment attached to artificial lipidation motifs were stably integrated into the *ste18* locus of an **a**-type haploid yeast chromosome, resulting in MC-FC and MC-FN yeast strains (Table 1). For the candidate proteins, autonomous replication plasmids for the expression of the four different Z variants (Z_WT_, Z_K35A_, Z_I31A_ and Z_955_) fused to Gγ_cyto_ (Gγ_cyto_-Y_1_) (pGK413-Gγ-EZWT, pGK413-Gγ-EZK35A, pGK413-Gγ-EZI31A and pGK413-Gγ-EZ955) (Table 2) were introduced into the MC-FC and MC-FN yeast cells (Fig. 3A and Fig. S2A). Flow cytometric analysis of the transformants was conducted after incubation in medium containing the α-alpha-cell mating pheromone (α-factor) (Fig. S1; **left**). The engineered yeast strains expressing the Gγ_cyto_-Z_WT_ and Gγ_cyto_-Z_K35A_ fusion proteins as candidates slightly induced the transcription of *GFP* reporter genes via interaction with the membrane-associated Fc fragment, although the fluorescence levels were extremely low (Fig. 3B and Fig. S2B). In mating selection with intact α-type yeast cells (Fig. S1; **right**), the strains expressing Gγ_cyto_-Z_WT_ and Gγ_cyto_-Z_K35A_ exhibited specific but negligible cell growth on selective medium (Fig. 3C and Fig. S2C). In both *GFP* transcription assays and mating growth selection, interactions of Gγ_cyto_-Z_I31A_ (very low affinity for Fc) and Gγ_cyto_-Z_955_ (negative control) with the membrane-associated Fc fragment were not detected. These results showed that the previous protocol was not sufficient to screen the interactions between membrane-associated target ‘X’ and candidate ‘Y_1_’-fused Gγ_cyto_ proteins.

### PPI-detecting Gγ recruitment system for the selection of candidate proteins interacting with membrane protein targets

Next, we tested the new protocol, in which we changed the method used to introduce the Gγ_cyto_-Y_1_ candidate genes. The DNA cassettes for cytosolic expression of the Gγ_cyto_-fused candidate Z variants (Z_WT_, Z_K35A_, Z_I31A_ and Z_955_) as a library were stably integrated into the MC-FC and MC-FN yeast chromosomes, generating FC(FN)-GW, FC(FN)-GK, FC(FN)-GI and FC(FN)-G9 strains (Table 1) (Fig. 3D and Fig. S2D). The engineered yeast strains chromosomally harboring Gγ_cyto_-Z_WT_ and Gγ_cyto_-Z_K35A_ genes showed apparent fluorescence in the *GFP* transcription assays (Fig. 3E and Fig. S2E). Similarly, in the mating selection, the same strains grew well on the selective medium (Fig. 3F and Fig. S2F). Compared with the Gpa1p-derived N-terminal lipidation motif, the C-terminally attached Ste18p lipidation motif was likely slightly favorable for PPI detection due to a reduction in accessibility between the membrane-associated Fc fragment and the Gγ_cyto_-fused Z domain (Fig. 3D–F and Fig. S2D–F). These results were clearly different from those following expression of Gγ_cyto_-fused candidate ‘Y_1_’ using autonomous replicating plasmids (Fig. 3A–C and Fig. S2A–C).

### Competitive selection of affinity-enhanced protein variants interacting with membrane protein targets using a previous protocol

**Figure 2:**
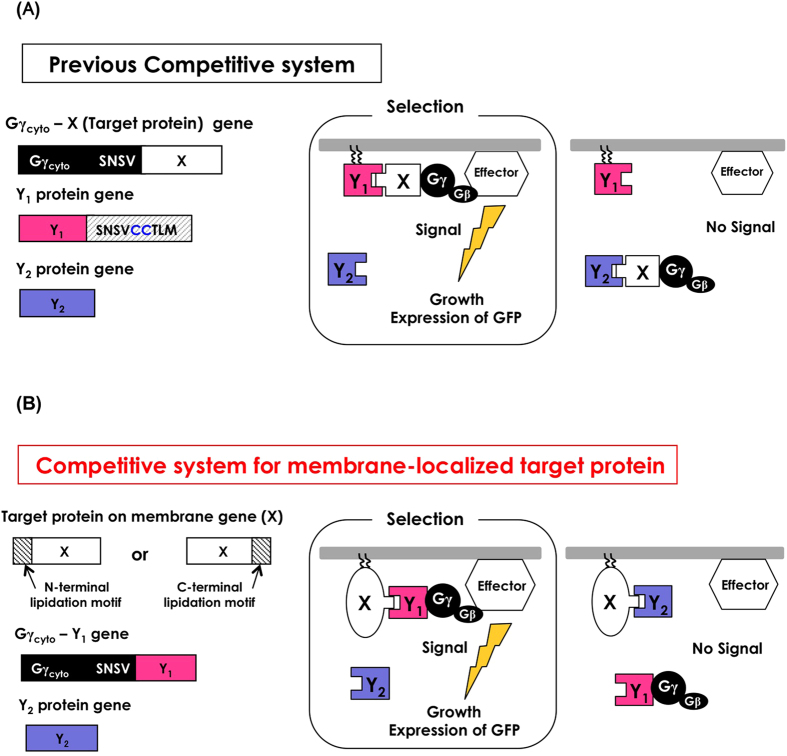
Schematic diagram of competitor-introduced Gγ recruitment systems to screen affinity-altered protein variants for cytosolic or membrane target proteins. (**A**) Schematic outline of the previously established competitor-introduced Gγ recruitment system for cytosolic target proteins. Target protein ‘X’ should be expressed as a fusion with Gγ_cyto_ in the cytosol. Protein ‘Y_1_’ should be anchored to the plasma membrane, whereas ‘Y_2_’ should be expressed in the cytosol. By establishing ‘Y_1_’ and ‘Y_2_’ as the parental (known) proteins originally bound to target ‘X’ and the candidate variant proteins, respectively, ‘Y_1_’ and ‘Y_2_’ compete to bind to target ‘X.’ When ‘X’ has higher affinity for ‘Y_2_,’ G-protein signaling is prevented due to the inability of Gγ_cyto_ to migrate to the plasma membrane. When ‘X’ has higher affinity for ‘Y_1_,’ G-protein signaling is transmitted into the yeast cells and invokes the mating process. Thus, affinity-enhanced proteins or affinity-attenuated proteins can be screened in a specific manner. (**B**) Schematic outline of competitor-introduced Gγ recruitment system for membrane protein targets. Target protein ‘X’ is a transmembrane or membrane-associated protein. Protein ‘Y_1_’ should be fused to Gγ_cyto_, whereas ‘Y_2_’ should be expressed in the cytosol. By establishing ‘Y_1_’ and ‘Y_2_’ as the parental (known) proteins originally bound to the membrane target ‘X’ and the candidate variant proteins, respectively, ‘Y_1_’ and ‘Y_2_’ compete to bind to target ‘X.’ When ‘X’ has a higher affinity for ‘Y_2_,’ G-protein signaling is prevented due to the inability of Gγ_cyto_ to migrate to the plasma membrane. When ‘X’ has higher affinity for ‘Y_1_’ fused to Gγ_cyto_, G-protein signaling is transmitted into the yeast cells and initiates the mating process.

Previously, we established the competitor-introduced Gγ recruitment system for selective screening of protein variants that exceed a specified affinity threshold^41^ (Fig. 2A). In the conventional Gγ recruitment system, additional expression of a cytosolic parental (known) protein (Y_2_) that binds to Gγ_cyto_-fused target protein ‘X’ competes with artificially membrane-associated protein variants as a candidate library (Y_1_), thereby permitting the selective screening of affinity-enhanced protein variants (Fig. 2A).

To test whether the previous competitor-introduced Gγ recruitment system allows for the use of membrane proteins as target ‘X’ (Fig. 2B and S3A), we consistently used the membrane-associated Fc fragment and the Gγ_cyto_-fused Z variants as target ‘X’ and candidate ‘Y_1_’ proteins, respectively (Figs. 4A and S4A). Z_I31A_ (low affinity for Fc; 8.0 × 10^3^ M^−1^) was utilized as the model of the competitive parental ‘Y_2_’ protein (Figs. 4B and S4B). Therefore, the Z_WT_ and Z_K35A_ candidate proteins (Y_1_), with higher affinities, should have outcompeted the interaction between membrane-associated Fc (X) and cytosolic Z_I31A_ (Y_2_), recovering the signaling in the system (Fig. S3A). In the previous system, the DNA cassette for Z_I31A_ expression as a competitor ‘Y_2_’ protein in the cytosol was stably integrated into the yeast chromosome of MC-FC, in which the C-terminally membrane-associated Fc fragment (X) (with the Ste18p lipidation motif) was expressed, generating an FC-I strain (Table 1). Autonomous replication plasmids for expression of the Gγ_cyto_-fused Z variants as candidate ‘Y_1_’ (pGK413-Gγ-EZWT, pGK413-Gγ-EZK35A, pGK413-Gγ-EZI31A and pGK413-Gγ-EZ955) (Table 2) were then introduced into the FC-I strain. However, both flow cytometric analysis and mating selection were barely able to detect the interactions between the membrane-associated Fc fragment (target ‘X’) and the Gγ_cyto_-fused Z variants (candidate ‘Y_1_’) relative to the interactions between the membrane-associated Fc fragment and cytosolic Z_I31A_ in all transformants (Fig. 4B). Additionally, when using an FN-I strain chromosomally expressing an N-terminally membrane-associated Fc fragment (X) (with a Gpa1p lipidation motif) and competitive Z_I31A_ protein (Y_2_) (Table 1), the transformants in which the candidate autonomous plasmids were introduced to express the Gγ_cyto_-fused Z variants (Y_1_) provided similar results to the C-terminally membrane-associated Fc fragment (Fig. S4B). These results showed that the previous system was unable to screen the interactions between membrane-associated target ‘X’ and candidate ‘Y_1_’-fused Gγ_cyto_ proteins relative to the interactions between membrane target ‘X’ and the cytosolic ‘Y_2_’ competitor.

### Competitor-introduced Gγ recruitment system that specifically selects affinity-enhanced protein variants interacting with membrane protein targets

Similar to what was described in the previous section, we attempted to change the protocol by introducing the expression cassettes for Gγ_cyto_-Y_1_ candidate genes into the competitor-introduced Gγ recruitment system (Figs. 4C and S4C). As competitive parental ‘Y_2_’ proteins, the genes for expressing the four different Z variants (Z_WT_, Z_K35A_, Z_I31A_ and Z_955_) in the cytosol were integrated into the MC-FC yeast chromosome (also expressing the C-terminally membrane-associated Fc fragment with the Ste18p lipidation motif as target ‘X’), generating FC-W, FC-K, FC-I and FC-9. The DNA cassettes for expressing the Gγ_cyto_-fused candidate Z variants as model library Y_1_ proteins were then stably integrated into the chromosome of the four yeast strains, generating 16 engineered yeast strains (FC-GWW through FC-G99; Table 1) (Fig. 4C).

Both flow cytometric analysis and mating selection revealed the interactor combinations between membrane-associated Fc and the Gγ_cyto_-fused Z variants serving as candidate ‘Y_1_’ proteins, with higher affinities than when the cytosolic Z variants served as competitor ‘Y_2_’ proteins (e.g., Y_1_ and Y_2_: Z_WT_ and Z_K35A_; Z_WT_ and Z_I31A_; and Z_K35A_ and Z_I31A_), although the very weak interactions between Fc and Gγ_cyto_-fused Z_I31A_ (Y_1_ and Y_2_: Z_I31A_ and Z_955_) could not be detected (Fig. 4D,E). These results clearly showed that the strains recovered signal transmission only when interactions between the membrane-associated Fc fragment (target ‘X’) and the Gγ_cyto_-fused Z variants (candidate ‘Y_1_’) overcame the competitive interactions between Fc (target ‘X’) and the cytosolic Z variants (competitor ‘Y_2_’). Additionally, when using a strain chromosomally expressing the N-terminally membrane-associated Fc fragment (X) (with the Gpa1p lipidation motif) (FN-GWW through FN-G99; Table 1), similar results were obtained (Fig. S4C–E).

Thus, Gγ_cyto_-fused ‘Y_1_’ candidate proteins should be stably integrated into the yeast chromosome to specifically select the affinity-enhanced protein variants against membrane-associated protein ‘X’ in the competitor-introduced Gγ recruitment system. This modification of the method made the competitor-introduced Gγ recruitment system able to screen affinity-enhanced protein variants by using membrane proteins as the target proteins.

### Competitive selection of affinity-attenuated protein variants interacting with membrane protein targets using a previous protocol

Previously, we also established a system that permits the selective screening of affinity-attenuated protein variants. In the conventional Gγ recruitment system, by setting the cytosolic protein (Y_2_) as the candidate library and the artificially membrane-associated protein (Y_1_) as the parental (known) competitor that binds to Gγ_cyto_-fused target protein ‘X,’ the system permits the selective screening of affinity-attenuated protein variants (Fig. 2A).

To test whether the previous competitor-introduced Gγ recruitment system allows for the use of membrane proteins as target ‘X’ (Fig. 2B and S3B), we consistently used the membrane-associated Fc fragment and the cytosolic Z variants as target ‘X’ and candidate ‘Y_2_’ proteins, respectively (Fig. 5A and S5A). Z_WT_ was utilized as the model of the competitive parental ‘Y_1_’ protein. Therefore, Gγ_cyto_-fused Z_WT_ (Y_1_) should have outcompeted the interactions between membrane-associated Fc (X) and the Z_K35A_, Z_I31A_ and Z_955_ candidate proteins (Y_2_), which have lower affinities, recovering the signaling in the system.

In the previous system, autonomous replication plasmids for expression of the Z variants in the cytosol as candidate ‘Y_2_’ proteins (pGK-LsZWTc, pGK-LsZK35Ac, pGK-LsZI31Ac and pGK-LsZ955c) (Table 2) were introduced into the FC-GW strain, which chromosomally expresses Fc-Ste18C as ‘X’ and Gγ_cyto_-Z_WT_ as competitor ‘Y_1_’ (Table 1). Both flow cytometric analysis and mating selection revealed the interactor combinations between membrane-associated Fc and the cytosolic Z variants serving as candidate ‘Y_2_’ proteins, whose affinities were lower than that of Gγ_cyto_-fused Z_WT_ as the competitor ‘Y_1_’ protein (Fig. 5B,C). Additionally, when using the FN-GW strain chromosomally expressing Gpa1N-Fc as ‘X’ and Gγ_cyto_-fused Z_WT_ as competitor ‘Y_1_’ (Table 1), the transformants in which the candidate autonomous plasmids were introduced to express the Z variants in the cytosol (Y_2_) provided similar results (Fig. S5B,C). In contrast to the affinity-enhanced system, these results showed that the previous competitor-introduced Gγ recruitment system was able to screen affinity-attenuated protein variants using membrane proteins as the target proteins.

### Demonstration of applicability of our system using intracellular domain of EGFR and Grb2

To demonstrate the applicability of our system, we selected the intracellular domain of EGFR (EGFR_cyto_), which contains a tyrosine kinase domain and tyrosine phosphorylation sites, and the adaptor protein Grb2 protein for the PPI pair^49^. In normal cells, binding of the epidermal growth factor (EGF) to the extracellular domain of EGFR leads to dimerization of the receptor and autophosphorylation of the receptor intracellular domain^50^,^51^. Grb2 binds to the phosphotyrosines of EGFR and links to the activation of subsequent intracellular signaling cascades^52^,^53^. In yeast, the intracellular domain of EGFR and its mutant derivatives have been often used to test the interaction with Grb2 protein^54^,^55^,^56^. To assay the interaction between EGFR and Grb2 in yeast, we used the intracellular domain of EGFR with L834R mutation (EGFR_L834R,cyto_; that is constitutively dimerized and activated even in the absence of EGF^49^,^57^) as the membrane protein by fusing several types of lipidation motifs at both the N-terminus (Gpa1p motif; Gpa1N) and the C-terminus (Ras1p motif; Ras1C and Ste18p motif; Ste18C). The Grb2 adaptor was fused to Gγ_cyto_ at the N-terminus and the C-terminus to test the accessibility between the membrane-associated EGFR_L834R,cyto_ and the cytosolic Gγ_cyto_-fused Grb2.

To express the membrane-associated EGFR_L834R,cyto_ protein, the genes encoding the EGFR_L834R,cyto_ attached to the artificial lipidation motifs (Ras1C, Ste18C and Gpa1N) were stably integrated into the *ste18* locus of an **a**-type haploid yeast chromosome, resulting in MC-ErC, MC-EsC and MC-EgN yeast strains (Table 1). For the candidate proteins, the DNA cassettes for cytosolic expression of the Gγ_cyto_-fused Grb2 at the N-terminus and the C-terminus (Gγ_cyto_-Grb2 and Grb2-Gγ_cyto_) were stably integrated into the MC-ErC, MC-EsC and MC-EgN yeast chromosomes, generating ErC-Ggrb(grbG), EsC-Ggrb(grbG) and EgN-Ggrb(grbG) (Table 1) (Fig. S6A,D). As a consequence of *GFP* transcription assays and mating selection, the engineered strains co-expressing the EGFR_L834R,cyto_ with C-terminal lipidation motifs (Ras1C and Ste18C) and the C-terminally Gγ_cyto_-fused Grb2 (Grb2-Gγ_cyto_) specifically showed apparent fluorescence and cell growth on the selective medium (Fig. S6A–F). The accessibility between the phosphotyrosines of membrane-associated EGFR_L834R,cyto_ and the SH2 domains of Grb2 or the distance of Gβγ_cyto_ complex from the membrane might have influenced the interactions of these proteins or to the subsequent membrane-anchored effector molecule^49^,^52^. Compared with the MC-ErC strain introducing the Grb2-Gγ_cyto_-expressing autonomous replicating plasmid (pGK413-Grb2-Gγ) (Table 2), the ErC-grbG strain that chromosomally expressed Grb2-Gγ_cyto_ was determinably more suitable for recovering the signaling (Fig. 6A–E).

To further test whether the competitor-introduced Gγ recruitment system that has designed to select the affinity-enhanced protein variants interacting with membrane target proteins is applicable to the intracellular domain of EGFR, we consistently used the membrane-associated EGFR_L834R,cyto_ and the Gγ_cyto_-fused Grb2 as membrane target ‘X’ and candidate ‘Y_1_’ proteins, respectively (Fig. 6F). Several Grb2 variants (Grb2, Grb2_E89K_ and Grb2_R86G_) with different affinities for the phosphotyrosines of EGFR were utilized for the competitive parental ‘Y_2_’ proteins (*K*_*a*_; Grb2 > Grb2_E89K_ > Grb2_R86G_)^58^.

Similar to what was described in the previous section, we tested the new protocol by chromosomally integrating the expression cassettes for Y_1_-Gγ_cyto_ candidate genes (Fig. 6F). As competitive parental ‘Y_2_’ proteins, the genes for expressing the three different Grb2 variants (Grb2, Grb2_E89K_ and Grb2_R86G_) in the cytosol were integrated into the ErC-grbG yeast chromosome (also co-expressing the membrane-associated EGFR_L834R,cyto_ with the Ras1p lipidation motif as target ‘X’ and the Grb2-Gγ_cyto_ fusion protein as candidate ‘Y_1_-Gγ_cyto_’), generating ErC-grbG-grb, ErC-grbG-E89K and ErC-grbG-R86G (Table 1). ErC-grbG-LEU yeast strain never expressing any competitor proteins was also generated as positive control (Table 1).

Both flow cytometric analysis and mating selection displayed the consistent results with the Z variants as expected (Fig. 6G,H). When using the strains respectively expressing Grb2_E89K_ and Grb2_R86G_ as the competitive parental ‘Y_2_’ proteins (ErC-grbG-E89K and ErC-grbG-R86G), the Gγ_cyto_-fused Grb2 expressed as candidate ‘Y_1_’ (Grb2-Gγ_cyto_) predictably recovered the signaling in accordance with the order of difference in the affinity strengths between the competitive proteins and the candidate proteins. Similarly, the strain co-expressing the same Grb2 protein as the candidate ‘Y_1_’ and the parental ‘Y_2_’ proteins (ErC-grbG-grb) barely showed GFP fluorescence and cell growth on the selective medium. Thus, we demonstrated that our systems were applicable to the membrane protein, which linked to the cellular states involved in various diseases.

### Discussion

In this study, we found that the previously established Gγ recruitment systems^41^,^42^ were basically unable to utilize membrane proteins as target protein ‘X.’ The new systems described here successfully enable the use of membrane proteins as target ‘X,’ both in the conventional (for screening of PPI candidate ‘Y_1_’ proteins) and competitor-introduced (for screening of affinity-enhanced candidate ‘Y_1_’ protein variants) Gγ recruitment systems. In the new systems, only the protocol for expression of Gγ_cyto_-fused candidate ‘Y_1_’ proteins was changed: instead of autonomous replicating plasmids, chromosomal integration was employed. These new systems are therefore very simple but highly useful. The results of the intracellular domain of EGFR and Grb2 interaction showed that our Gγ recruitment systems could be exploited as a convenient heterologous system to discern the strong binders to the phosphotyrosines in the intracellular domain of EGFR, and therefore would provide the basis for studying other receptor tyrosine kinases as well. In this manner, the screening of binding partners and affinity-enhanced variants targeted to the inner domains of these membrane proteins has great potential for applications in the treatment of human diseases.

**Figure 3:**
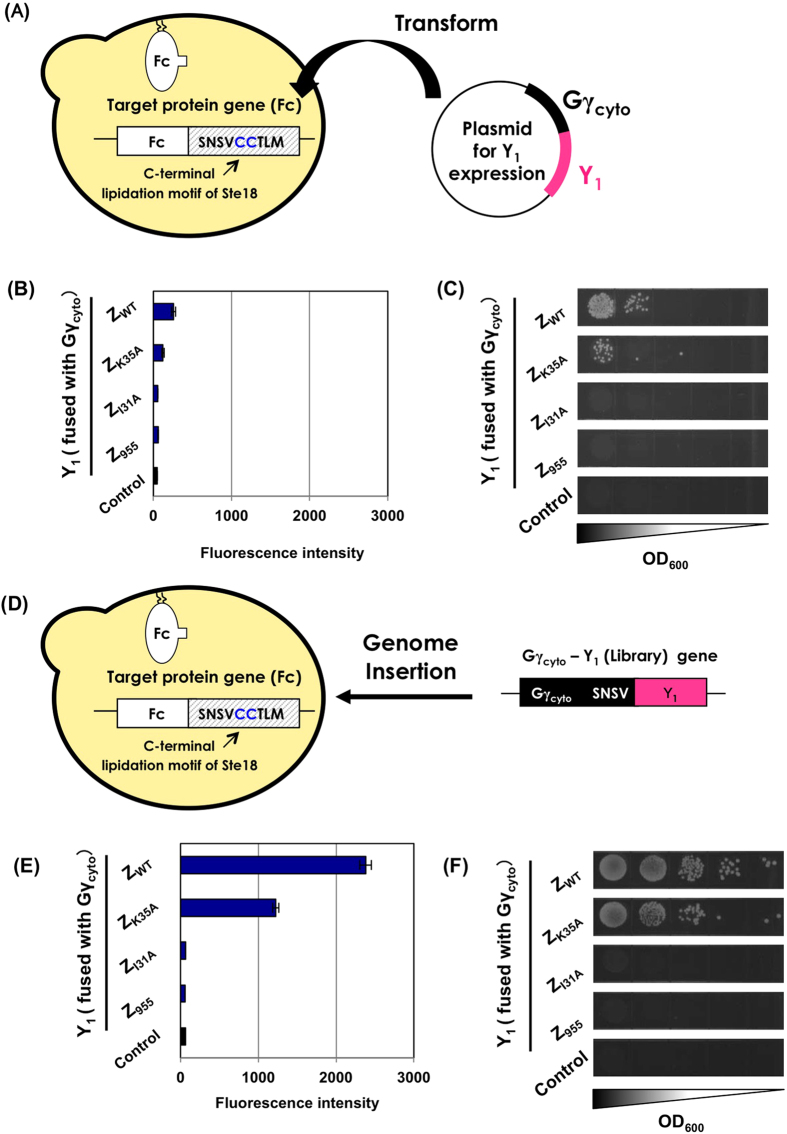
Selection of Z variants binding to a membrane-associated target Fc protein using previous and new Gγ recruitment systems. (**A**) Previous Gγ recruitment system for membrane proteins as targets. (**B,C**) Flow cytometric analyses and mating growth assay. The fluorescence and growth intensities of the engineered strains expressing C-terminally membrane-associated Fc via stable integration into the yeast chromosome as well as cytosolic Z variants fused to Gγ_cyto_ ‘Y_1_’ via autonomous replication plasmids. The control yeast shows the strain without the expression of ‘Y_1_’ fused to Gγ_cyto_ (transformed with pGK413 mock vector). (**D**) New Gγ recruitment system for membrane proteins as targets. (**E,F**) Flow cytometric analyses and mating growth assay. The fluorescence and growth intensities of the engineered strains expressing C-terminally membrane-associated Fc and cytosolic Z variants fused to Gγ_cyto_ via stable integration into the yeast chromosome. The control yeast shows the strain without the expression of ‘Y_1_’ fused to Gγ_cyto_ (MC-FC in Table 1).

**Figure 4:**
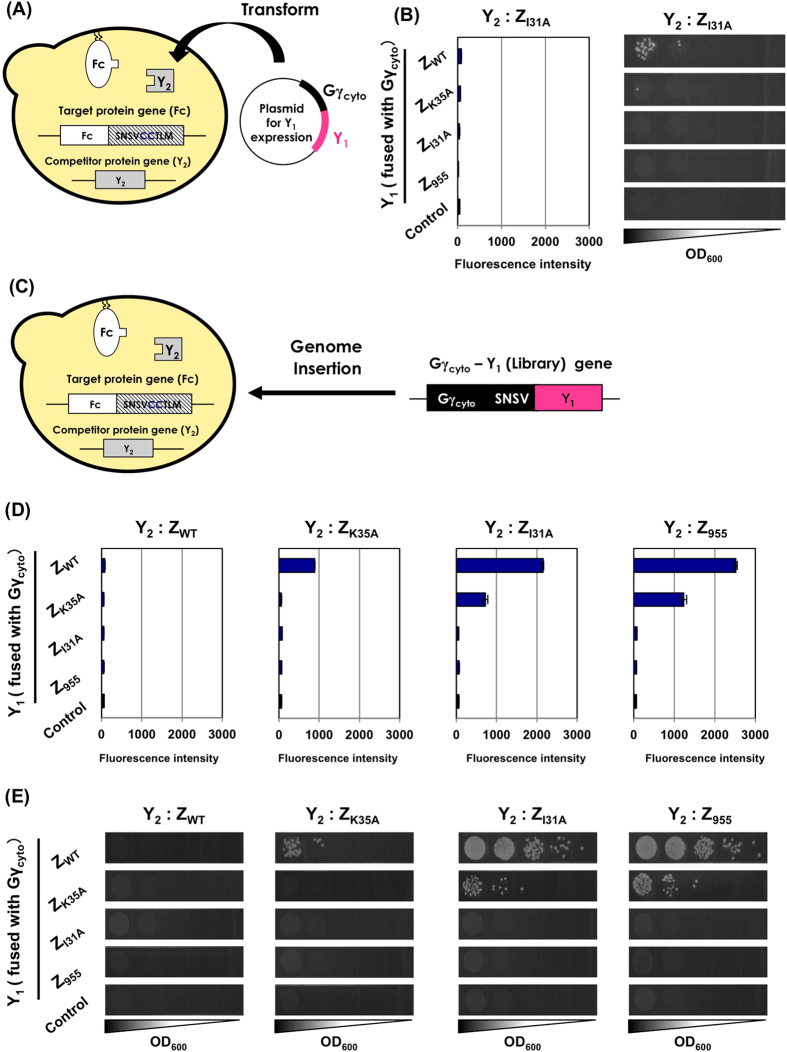
Competitive selection of Z variants with higher affinities for membrane-associated target Fc using previous and new methods for affinity-enhanced systems. (**A**) Previous affinity-enhanced system for membrane proteins as targets. (**B**) Flow cytometric analyses and mating growth assay. The fluorescence and growth intensities of the engineered strains expressing C-terminally membrane-associated Fc and competitor Z_I31A_ as cytosolic ‘Y_2_’ via stable integration into the yeast chromosome as well as cytosolic Z variants ‘Y_1_’ fused to Gγ_cyto_ via autonomous replication plasmids. Control yeast strains lacked the expression of ‘Y_1_’ fused to Gγ_cyto_ (transformed with pGK413 mock vector). (**C**) New affinity-enhanced system for membrane proteins as target. (**D,E**) Flow cytometric analyses and mating growth assay. The fluorescence and growth intensities of the engineered strains expressing C-terminally membrane-associated Fc, competitor cytosolic Z variants ‘Y_2_’ and cytosolic Z variants ‘Y_1_’ fused to Gγ_cyto_ via stable integration into the yeast chromosome. The control yeast shows the strain without the expression of ‘Y_1_’ fused to Gγ_cyto_.

**Figure 5:**
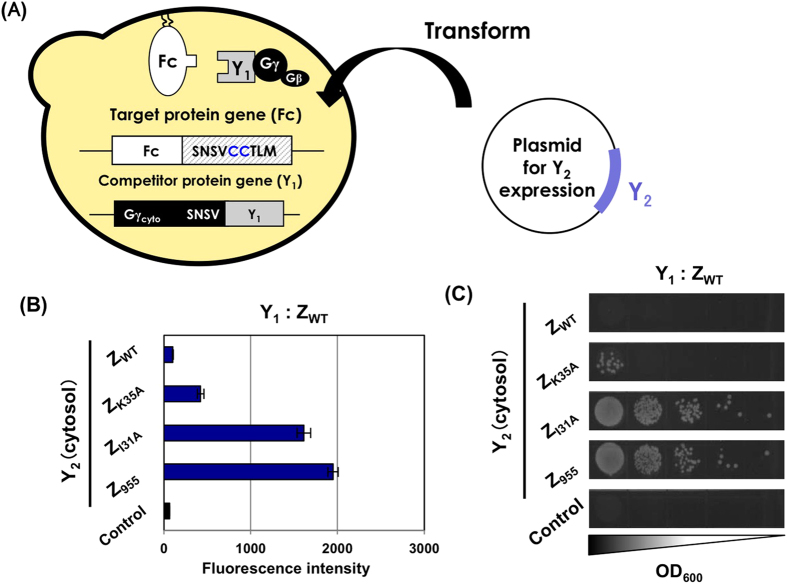
Competitive selection of Z variants with lower affinities for membrane-associated target Fc using the previous affinity-attenuated system. (**A**) Previous affinity-attenuated system for membrane proteins as targets. (**B,C**) Flow cytometric analyses and mating growth assay. The fluorescence and growth intensities of the engineered strains expressing C-terminally membrane-associated Fc and competitor Z_WT_ as cytosolic ‘Y_1_’ fused to Gγ_cyto_ via stable integration into the yeast chromosome and cytosolic Z variants ‘Y_2_’ via autonomous replication plasmids. The control yeast shows the strain without the expression of ‘Y_1_’ fused to Gγ_cyto_ and cytosolic Z variants ‘Y_2_.’

**Figure 6:**
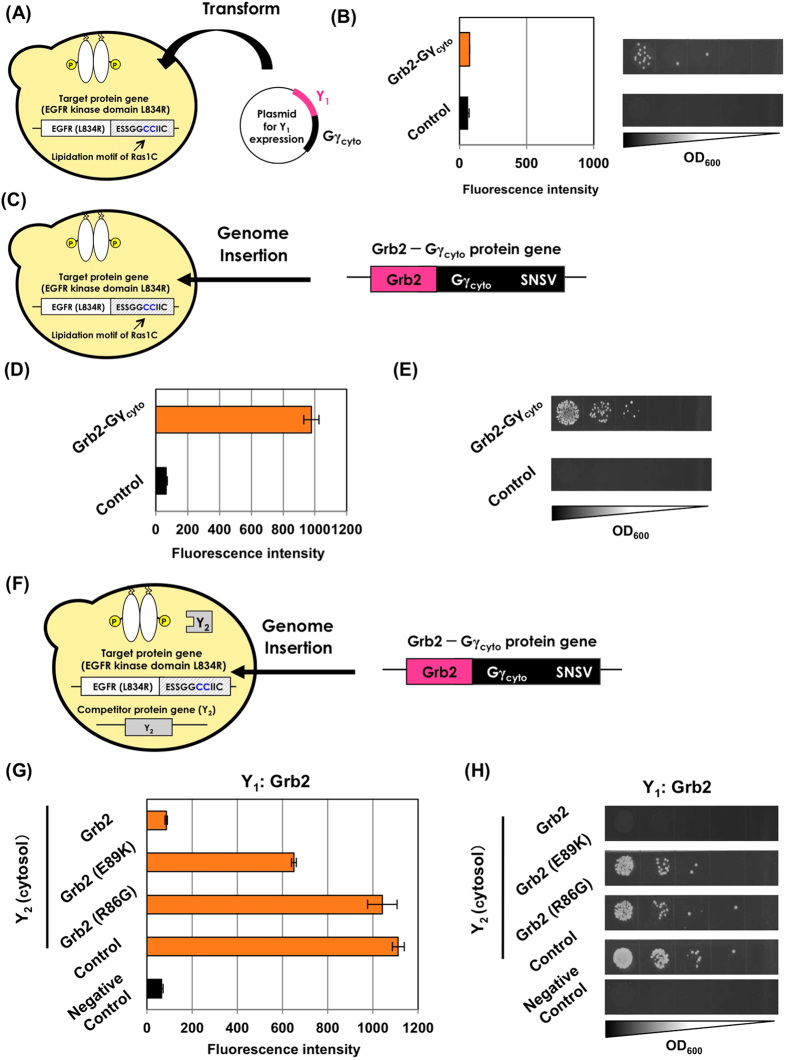
Competitive selection of Grb2 for membrane-associated intracellular domain of EGFR. (**A**) Previous Gγ recruitment system for intracellular domain of EGFR as the membrane target. (**B**) Flow cytometric analyses and mating growth assay. The fluorescence and growth intensities of the engineered strains expressing C-terminally membrane-associated intracellular domain of EGFR L834R mutant (EGFR_L834R,cyto_) via stable integration into the yeast chromosome as well as cytosolic Grb2 fused to Gγ_cyto_ ‘Y_1_’ (Grb2-Gγ_cyto_) via autonomous replication plasmids. The control yeast shows the strain without the expression of Grb2-Gγ_cyto_ (transformed with pGK413 mock vector). (**C**) New Gγ recruitment system for intracellular domain of EGFR as the membrane target. (**D,E**) Flow cytometric analyses and mating growth assay. The fluorescence and growth intensities of the engineered strains expressing C-terminally membrane-associated EGFR_L834R,cyto_ and cytosolic Grb2-Gγ_cyto_ via stable integration into the yeast chromosome. The control yeast shows the strain without the expression of Grb2-Gγ_cyto_ (MC-ErC in Table 1). (**F**) New affinity-enhanced system for intracellular domain of EGFR as the membrane target. (**G,H**) Flow cytometric analyses and mating growth assay. The fluorescence and growth intensities of the engineered strains expressing C-terminally membrane-associated EGFR_L834R,cyto_, competitor cytosolic Grb2 variants ‘Y_2_’ (Grb2, Grb2_E89K_ and Grb2_R86G_) and cytosolic Grb2 ‘Y_1_’ fused to Gγ_cyto_ (Grb2-Gγ_cyto_) via stable integration into the yeast chromosome. The control yeast shows the strain without the expression of any competitive ‘Y_2_’ proteins (ErC-grbG-LEU in Table 1). The negative control yeast shows the strain without the expression of ‘Y_1_’ fused to Gγ_cyto_.

Previously, we demonstrated that Gγ recruitment systems enabled extremely reliable screening that could completely exclude false-positive candidates^41^,^42^. Generally, membrane yeast two-hybrid systems^30^,^31^,^33^,^34^ and protein fragment complementation assays^23^,^38^ sometimes exhibit background readouts^23^,^59^ due to the use of directly fused artificial transcription factors and automatic self-associations of the split proteins. These background readouts are a critical problem, even when they are negligible, especially in the case of growth screening using a large-scale library^23^. The exclusive selection in Gγ recruitment systems is made possible by using the signal transduction machinery, which requires the localization of Gβ/Gγ in *GFP* transcription assays and mating selection (Figs. 3, 4, 5). This extremely disciplined selection machinery makes Gγ recruitment systems worth using.

In the Gγ recruitment system that has designed for membrane proteins as the target, Z_I31A_ with extremely low affinity could not be detected in both cases of the flow cytometric analysis and the mating selection (Fig. 3). Due to the very low affinity between Z_I31A_ and the Fc region (8.0 × 10^3^ M^−1^), the migration of Gγ_cyto_ to the membrane was likely insufficient for the recovering of the signal transduction. This affinity (8.0 × 10^3^ M^−1^) seems to be less than a lower limit of our present system, although it is unlikely that a protein mutant exhibiting such extremely low affinity would be required.

From the perspective of screening for a target membrane protein ‘X’, the new methods that chromosomally integrate the DNA cassettes expressing Gγ_cyto_-fused candidate ‘Y_1_’ proteins might have a handicap in constructing a library. Specifically, the transformation efficiencies of homologous integrations into the yeast chromosome are commonly 10^1^–10^2^ fold lower than those of autonomous replicating plasmids (approximately 10^5^–10^6^ cfu/μg)^60^,^61^,^62^. Therefore, constructing a large-scale library might require a little ingenuity to increase the transformation efficiencies, such as via the use of large amounts of DNA, the electroporation method^61^,^63^, the spheroplasting method^64^, and use of I-SceI meganuclease^65^. Even allowing for this additional effort, however, the conventional Gγ recruitment system is a powerful tool because of its extremely reliable selection of binding partners. In addition, the competitor-introduced Gγ recruitment system, which allows for the specific screening of affinity-enhanced protein variants (specifically excluding protein variants showing equal or lower affinities^41^), is valuable as a unique and irreplaceable growth selection technique.

A similar approach for screening for affinity-attenuated protein variants among membrane proteins serving as target ‘X’ made it possible to apply the previous method using autonomous replicating plasmids to express the candidate ‘Y_2_’ in the cytosol (Fig. 5). We believe that the unstable expression of ‘Y_1_’-fused Gγ_cyto_ using autonomous replicating plasmids rendered the Gγ recruitment system useless. Because it has been reported that plasmid retentions become unstable during signal-promoted states^66^, ‘Y_1_’-fused Gγ_cyto_ might be more affected by this unstable plasmid retention than cytosolic ‘Y_2_’ is. In any event, the chromosomal expression of ‘Y_1_’-fused Gγ_cyto_ is favorable in our Gγ recruitment systems.

In summary, new Gγ recruitment systems make it possible for membrane proteins to be target protein ‘X.’ These systems permit reliable and specific screens for binding partners and affinity-enhanced protein variants. We envision that our selection method will provide a powerful, broadly applicable tool for studying biological processes, creating new opportunities to develop new drugs targeting a wide range of membrane-associated proteins and inner domains of transmembrane proteins.

## Methods

### Strains and media

The genotypes of *Saccharomyces cerevisiae* BY4741^67^, MC-F1^43^, and BY4742^67^ and the other recombinant strains used in this study are provided in Table 1. The yeast strains were grown in YPD medium containing 1% (w/v) yeast extract, 2% peptone and 2% glucose or in SD medium containing 0.67% yeast nitrogen base without amino acids (BD Diagnostic Systems, Sparks, MD, USA) and 2% glucose. The SD medium was supplemented with amino acids and nucleotides (20 mg/L histidine, 60 mg/L leucine, 20 mg/L methionine, or 20 mg/L uracil), as required by the auxotrophic strains. Agar (2%; w/v) was added to the medium to produce YPD and SD solid media.

### Construction of plasmids

All plasmids and primers used in this study are listed in Table 2 and Table S1. Plasmids inserting lipidation motifs were constructed as follows. The fragments of the *PGK1* promoter (*P*_*PGK1*_) fused to the lipidation motif of Gpa1p (9 a.a. of N-terminus) and the multi-cloning site were amplified from pGK425^68^ using primer 1, primer 2 and primer 3 and inserted into the *Xho*I-*Bgl*II sites of the autonomous replication plasmid pGK425^68^, yielding plasmid pGK425-Gpa1N. The fragments of the *PGK1* promoter fused to the lipidation motif of Ste18p (9 a.a. of C-terminus) and the multi-cloning site were amplified from pGK425^68^ using primer 1, primer 4 and primer 5 and inserted into the *Xho*I-*Bgl*II sites of the autonomous replication plasmid pGK425^68^, yielding plasmid pGK425-Ste18C. The fragments of the *PGK1* promoter fused to the lipidation motif of Ras1p (10 a.a. of C-terminus) and the multi-cloning site were amplified from pGK425^68^ using primer 1, primer 6 and primer 7 and inserted into the *Xho*I-*Bgl*II sites of the autonomous replication plasmid pGK425^68^, yielding plasmid pGK425-Ras1C.

The plasmids used for the expression of the Fc fragment on the membrane were constructed as follows. The fragments encoding the Fc protein were amplified from pUMGP-GγMFcH^42^ using primers 8 and 9 or primers 10 and 11 and inserted into the *Sal*I-*BamH*I sites of the autonomous replication plasmid pGK425-Gpa1N or pGK425-Ste18C, yielding pGK425-Gpa1N-Fc and pGK425-Fc-Ste18C, respectively. The cassettes for expression of the membrane-associated Fc protein for integration at the *ste18* locus on the yeast chromosome were then amplified from pGK425-Gpa1N-Fc or pGK425-Fc-Ste18C using primer 12 and primer 13 and inserted into the *Xho*I sites of pGK426-GPTK^42^ using an In-Fusion HD Cloning Kit (Clontech Laboratories – Takara Bio, Shiga, Japan), yielding pUMGPTK-Gpa1N-Fc and pUMGPTK-Fc-Ste18C, respectively.

The plasmids used for the expression of the Gγ_cyto_-Z domain variants in the cytosol were constructed as follows. The fragment encoding Gγ lacking the lipidation sites (Gγ_cyto_) was amplified from pUMGP-GγMFcH^42^ using primer 14 and primer 15. The fragments encoding the Z variants (Z_WT_, Z_K35A_, Z_I31A_ and Z_955_) were amplified from pGK-LsZWTc, pGK-LsZK35Ac, pGK-LsZI31Ac and pGK-LsZ955c^41^ using primer 16 and primer 17. The fusion fragments encoding the Gγ_cyto_-Z domain were then amplified from these two fragments by overlap PCR using primer 14 and primer 17 and inserted into the *Sal*I-*EcoR*I sites of the autonomous replication plasmid pGK413^68^, yielding plasmids pGK413-Gγ-EZWT, pGK413-Gγ-EZK35A, pGK413-Gγ-EZI31A and pGK413-Gγ-EZ955, respectively. Subsequently, the cassettes for expression of the Gγ_cyto_-Z variants for integration at the *his3* locus on the yeast chromosome were constructed as follows. The fragment containing the *STE18* promoter (*P*_*STE18*_) and the gene encoding Gγ_cyto_ were amplified from pUMGP-GγMFcH^42^ using primer 18 and primer 19 and inserted into the *Xho*I-*Nhe*I sites of pGK426^68^, yielding plasmid pUSTE18p-Gγcyto. The fragment encoding *HIS3* terminator (*T*_*HIS3*_) was amplified from the BY4741 genome using primer 20 and primer 21 and inserted into the *Not*I-*Sac*I sites of pUSTE18p-Gγcyto, yielding plasmid pUSTE18p-Gγcyto-HIS3t. Finally, the fragments encoding the Z variants were amplified from pGK-LsZWTc, pGK-LsZK35Ac, pGK-LsZI31Ac and pGK-LsZ955c^41^ using primer 22 and primer 23 and inserted into the *Sal*I-*BamH*I sites of pUSTE18p-Gγcyto-HIS3t, yielding plasmids pUSTE18p-Gγcyto-ZWT-HIS3t, pUSTE18p-Gγcyto-ZK35A-HIS3t, pUSTE18p-Gγcyto-ZI31A-HIS3t and pUSTE18p-Gγcyto-Z955-HIS3t, respectively.

The cassettes for expression of the cytosolic Z variants as competitors for integration upstream of the *HOP2* gene locus (*P*_*HOP2*_: *HOP2* promoter region) on the yeast chromosome were constructed as follows. The fragments encoding *P*_*HOP2*_ were amplified using primer 24 and primer 25 and inserted into the *Not*I-*Sac*I sites of pGK-LsZWTc, pGK-LsZK35Ac, pGK-LsZI31Ac and pGK-LsZ955c^41^, yielding plasmids pGK-LsZWTc-HOP, pGK-LsZK35Ac-HOP, pGK-LsZI31Ac-HOP and pGK-LsZ955c-HOP, respectively.

The plasmids used for the expression of the intracellular domain of EGFR L834R mutant (EGFR_L834R,cyto_) on the membrane were constructed as follows. The fragments encoding the intracellular domain of EGFR_L834R,cyto_ mutant were amplified from the B1U-GL^49^ genome using primers 26 and 27 or primers 28 and 29 and inserted into the *Sal*I-*Mlu*I sites of pGK425-Gpa1N, pGK425-Ste18C and pGK425-Ras1C, yielding pGK425-Gpa1N-EGFR(LR), pGK425-EGFR(LR)-Ste18C and pGK425-EGFR(LR)-Ras1C, respectively. The cassettes for expression of the membrane-associated EGFR_L834R,cyto_ for integration at the *ste18* locus on the yeast chromosome were then amplified from pGK425-Gpa1N-EGFR(LR), pGK425-EGFR(LR)-Ste18C and pGK425-EGFR(LR)-Ras1C using primer 12 and primer 13 and inserted into the *Xho*I sites of pGK426-GPTK^42^ using an In-Fusion HD Cloning Kit, yielding pUMGPTK-Gpa1N-EGFR(LR), pUMGPTK-EGFR(LR)-Ste18C and pUMGPTK-EGFR(LR)-Ras1C, respectively.

The plasmids used for the expression of the Grb2-Gγ_cyto_ in the cytosol were constructed as follows. The fragment encoding the Grb2-Gγ_cyto_ was amplified from B1U-GL^49^ using primer 30 and primer 31 and inserted into the *Sal*I-*EcoR*I sites of the autonomous replication plasmid pGK413^68^ using an In-Fusion HD Cloning Kit, yielding plasmid pGK413-Grb2-Gγ. Subsequently, the cassettes for expression of the Grb2-Gγ_cyto_ for integration at the *his3* locus on the yeast chromosome were constructed as follows. The fragment containing the *STE18* promoter (*P*_*STE18*_) was amplified from pUMGP-GγMFcH^42^ using primer 32 and primer 33 and inserted into the *Xho*I-*Nhe*I sites of pGK416^68^, yielding plasmid Ste18p-416. The fragment containing the gene encoding Gγ_cyto_ were amplified from pUMGP-GγMFcH^42^ using primer 34 and primer 35 and inserted into the *Xba*I-*EcoR*I sites of Ste18p-416, yielding plasmid pUSTE18p-c-Gγcyto. The fragment encoding *HIS3* terminator (*T*_*HIS3*_) was amplified from the BY4741 genome using primer 20 and primer 21 and inserted into the *Not*I-*Sac*I sites of pUSTE18p-c-Gγcyto, yielding plasmid pUSTE18p-c-Gγcyto-HIS3t. Finally, the fragment encoding Grb2 was amplified from pGK413-Grb2-Gγ using primer 36 and primer 37 and inserted into the *Nhe*I-*Xma*I sites of pUSTE18p-c-Gγcyto-HIS3t, yielding plasmid pUSTE18p-Grb2-Gγcyto-HIS3t.

The plasmids used for the expression cassettes of the Gγ_cyto_-Grb2 for integration at the *his3* locus on the yeast chromosome were constructed as follows. The fragment encoding Grb2 was amplified from pGK413-Grb2-Gγ using primer 38 and primer 39 and inserted into the *Nhe*I-*Xma*I sites of pUSTE18p-Gγcyto-HIS3t, yielding plasmid pUSTE18p-Gγcyto-Grb2-HIS3t.

The cassettes for expression of the cytosolic Grb2 variants as competitors for integration at the upstream of the *HOP2* gene locus (*P*_*HOP2*_: *HOP2* promoter region) on the yeast chromosome were constructed as follows. The fragments encoding *P*_*HOP2*_ were amplified using primer 24 and primer 25 and inserted into the *Not*I-*Sac*I sites of pGK415^68^, yielding plasmid pGK415-HOP2p. The fragment encoding Grb2 was amplified from pGK413-Grb2-Gγ using primers 38 and 39 and inserted into the *Sal*I-*Xma*I sites of pGK415-HOP2p using an In-Fusion HD Cloning Kit, yielding plasmid pGK415-Grb2-HOP2p. The fragment encoding Grb2_R86G_ mutant was amplified from pGK413-Grb2-Gγ using primers 40 and 42, primers 41 and 43 and the fragments encoding the Grb2_R86G_ mutant was amplified from these two fragments by overlap PCR using primer 40 and primer 41, and inserted into the *Sal*I-*Xma*I sites of pGK415-HOP2p using an In-Fusion HD Cloning Kit, yielding plasmid pGK415-Grb2_R86G_-HOP2p. The fragment encoding Grb2_E89K_ mutant was amplified from pGK413-Grb2-Gγ using primers 40 and 44, primers 41 and 45 and the fragments encoding the Grb2_E89K_ mutant was amplified from these two fragments by overlap PCR using primer 40 and primer 41, and inserted into the *Sal*I-*Xma*I sites of pGK415-HOP2p using an In-Fusion HD Cloning Kit, yielding plasmid pGK415-Grb2_E89K_-HOP2p.

### Construction of yeast strains

All strains used in this study are listed in Table 1. Integration of the DNA cassettes for expressing the membrane-associated Fc protein was achieved as follows. The DNA fragments containing *P*_*STE18*_*-P*_*PGK1*_*-Fc-Ste18C-T*_*PGK1*_*-kanMX4-T*_*STE18*_ and *P*_*STE18*_*-P*_*PGK1*_*-Gpa1N-Fc-T*_*PGK1*_*-kanMX4-T*_*STE18*_ were amplified from pUMGPTK-Fc-Ste18C and pUMGPTK-Gpa1N-Fc using primer 46 and primer 47. The amplified DNA fragments were then used to transform MC-F1^43^ using the lithium acetate method^69^. The transformants were selected on a YPD + G418 plate to yield MC-FC and MC-FN (Table 1).

Integration of the DNA cassettes for the Gγ_cyto_-Z domain variants (Z_WT_, Z_K35A_, Z_I31A_ and Z_955_) in the cytosol was achieved as follows. The DNA fragments containing *URA3-P*_*PGK1*_*-Gγ*_*cyto*_*-Z*_*WT*_*(-Z*_*K35A*_*, -Z*_*I31A*_ and *-Z*_*955*_*)-T*_*PGK1*_*-T*_*HIS3*_ were amplified from pUSTE18p-Gγcyto-ZWT(-ZK35A, -ZI31A and -Z955)-HIS3t using primer 48 (containing the homologous regions of the *HIS3* promoter) and primer 49. The amplified DNA fragments were used to transform MC-FC and MC-FN using the lithium acetate method^69^. The transformants were then selected on an SD-Ura plate (containing leucine, histidine and methionine) to yield FC-GW, FC-GK, FC-GI, and FC-G9 and FN-GW, FN-GK, FN-GI and FN-G9 (Table 1).

Integration of the DNA cassettes for expressing the Z variants (Z_WT_, Z_K35A_, Z_I31A_ and Z_955_) as competitors in the cytosol was achieved as follows. The DNA fragments containing *LEU2-P*_*PGK1*_*-Z*_*WT*_*(-Z*_*K35A*_*, -Z*_*I31A*_ and *-Z*_*955*_)-*T*_*PGK1*_*-P*_*HOP2*_ were amplified from pGK-LsZWTc(-LsZK35Ac, -LsZI31Ac and -LsZ955c)-HOP using primer 50 (containing the homologous regions of *P*_*HOP2*_ upstream) and primer 51. The amplified DNA fragments were used to transform FC-GW, FC-GK, FC-GI, and FC-G9 and FN-GW, FN-GK, FN-GI and FN-G9. The transformants were then selected on an SD-Leu/-Ura plate (containing histidine and methionine) to yield FC-GWW, FC-GWK, FC-GWI, and FC-GW9; FC-GKW, FC-GKK, FC-GKI, and FC-GK9; FC-GIW, FC-GIK, FC-GII, and FC-GI9; and FC-G9W, FC-G9K, FC-G9I, and FC-G99 as well as FN-GWW, FN-GWK, FN-GWI, and FN-GW9; FN-GKW, FN-GKK, FN-GKI, and FN-GK9; FN-GIW, FN-GIK, FN-GII, and FN-GI9; and FN-G9W, FN-G9K, FN-G9I, and FN-G99 (Table 1).

Integration of the DNA cassettes for expressing the membrane-associated intracellular domain of EGFR L834R mutant (EGFR_L834R,cyto_) was achieved as follows. The DNA fragments containing *P*_*STE18*_*-P*_*PGK1*_*-EGFR*_*L834R,cyto*_*-Ras1C-T*_*PGK1*_*-kanMX4-T*_*STE18*_*, P*_*STE18*_*-P*_*PGK1*_*-EGFR*_*L834R,cyto*_*-Ste18C-T*_*PGK1*_*-kanMX4-T*_*STE18*_ and *P*_*STE18*_*-P*_*PGK1*_*-Gpa1N-EGFR*_*L834R,cyto*_*-T*_*PGK1*_*-kanMX4-T*_*STE18*_ were amplified from pGK425-EGFR(LR)-Ras1C, pGK425-EGFR(LR)-Ste18C and pGK425-Gpa1N-EGFR(LR) using primer 46 and primer 47. The amplified DNA fragments were then used to transform MC-F1^43^ using the lithium acetate method^69^. The transformants were selected on a YPD + G418 plate to yield MC-ErC, MC-EsC and MC-EgN (Table 1).

Integration of the DNA cassettes for the Grb2-Gγ_cyto_ in the cytosol was achieved as follows. The DNA fragments containing *URA3-P*_*PGK1*_*-Grb2-Gγ*_*cyto*_*-T*_*PGK1*_*-T*_*HIS3*_ was amplified from pUSTE18p-Grb2-Gγcyto-HIS3t using primer 48 (containing the homologous regions of the *HIS3* promoter) and primer 49. The amplified DNA fragments were used to transform MC-ErC, MC-EsC and MC-EgN using the lithium acetate method^69^. The transformants were the selected on an SD-Ura plate to yield ErC-grbG, EsC-grbG and EgC-grbG (Table 1). Integration of the DNA cassettes for the Gγ_cyto_-Grb2 in the cytosol was achieved as follows. The DNA fragments containing *URA3-P*_*PGK1*_*-Gγ*_*cyto*_*-Grb2-T*_*PGK1*_*-T*_*HIS3*_ was amplified from pUSTE18p-Gγcyto-Grb2-HIS3t using primer 48 (containing the homologous regions of the *HIS3* promoter) and primer 49. The amplified DNA fragments were used to transform MC-ErC, MC-EsC and MC-EgN using the lithium acetate method^69^. The transformants were then selected on an SD-Ura plate to yield ErC-Ggrb, EsC-Ggrb and EgC-Ggrb (Table 1).

Integration of the DNA cassettes for expressing Grb2 variants (Grb2, Grb2_E89K_ and Grb2_R86G_) and positive control (no competitor expression) as the competitor in the cytosol was achieved as follows. The DNA fragments containing *LEU2-P*_*PGK1*_*-Grb2(-Grb2*_*E89K*_ and *-Grb2*_*R86G*_)-*T*_*PGK1*_*-P*_*HOP2*_ and *LEU2-P*_*PGK1*_*-T*_*PGK1*_*-P*_*HOP2*_ were amplified from pGK-LsGrb2(-LsGrb2(R89K) and -LsGrb2(R86G))-HOP and pGK415-HOP2p using primer 50 (containing the homologous regions of *P*_*HOP2*_ upstream) and primer 51. The amplified DNA fragments were used to transform ErC-grbG. The transformants were then selected on an SD-Leu/-Ura plate to yield ErC-grbG-grb, ErC-grbG-E89K, ErC-grbG-R86G and ErC-grbG-LEU (Table 1).

All transformants were obtained by introducing the autonomous replicating plasmids (Table 2) into these yeast strains using the lithium acetate method^69^.

### GFP reporter expression analysis

*GFP* reporter expression analysis basically followed previous methods^41^, with certain modifications. In the case of the previous method, the engineered yeast **a**-cells were grown in 5 mL of SD-His medium (for the PPI detection system), SD-His/-Leu medium (for the affinity-enhanced system) or SD-Leu/-Ura medium (for the affinity-attenuated system) at 30 °C overnight. The cultured cells were then inoculated in 2 mL of fresh SD-His, SD-His/-Leu or SD-Leu/-Ura medium containing 5 μM α-factor (Zymo Research, Orange, CA, USA) to obtain an initial OD_600_ of 0.1 (OD_600_ = 0.1). In the case of the new method, the engineered yeast **a**-cells were grown in 5 mL of YPD medium (for the PPI detection system and affinity-enhanced system) at 30 °C overnight. The cultured cells were then inoculated in 2 mL of fresh YPD medium containing 5 μM α-factor (Zymo Research, Orange, CA, USA) to obtain an initial OD_600_ of 0.1 (OD_600_ = 0.1). The expression of the *FIG1-EGFP* fusion reporter gene was then stimulated by growth at 30 °C for 6 hours.

The fluorescence intensities of the cultured cells were measured using a BD FACSCanto II flow cytometer equipped with a 488-nm blue laser (BD Biosciences, San Jose, CA, USA)^70^. The GFP fluorescence signal was specifically collected through a 530/30-nm band-pass filter. The mean fluorescence intensity was defined as the GFP-A mean of 10,000 cells. The data were analyzed using BD FACSDiva software (version 5.0, BD Biosciences).

### Mating growth spotting assay

The mating growth spotting assay basically followed a previous method^41^, with certain modifications. For the previous method, each engineered yeast **a**-cell was grown in 5 mL of SD-His media (for PPI detection system), SD-His/-Leu medium (for the affinity-enhanced system) or SD-Leu/-Ura medium (for the affinity-attenuated system) at 30 °C overnight and then cultivated in 5 mL of YPD medium with the mating partner, or the BY4742 α-cell^67^, at 30 °C for 3 hours. The initial OD_600_ of each haploid cell was set at 0.1 (OD_600_ = 0.1). For the new method, each engineered yeast **a**-cell was grown in 5 mL of YPD medium (for the PPI detection system and the affinity-enhanced system) at 30 °C overnight and then cultivated in 5 mL of YPD medium with the mating partner, or the BY4742 α-cell^67^, at 30 °C for 3 hours. The initial OD_600_ of each haploid cell was again set at 0.1 (OD_600_ = 0.1). After cultivation, the yeast cells were harvested, washed, and resuspended in distilled water. To quantify the mating ability of each strain, a dilution series of each yeast cell suspension was prepared (OD_600_ = 1.0, 0.1, 0.01, 0.001 and 0.0001), and 40 μL of each dilution was then spotted on a selective SD-Ura/Leu plate (lacking methionine, lysine and histidine; for the PPI detection system generated by the previous method), SD-Ura plate (lacking methionine, lysine, histidine and leucine; for the affinity-enhanced system generated by the previous method), SD-His plate (lacking methionine, lysine, leucine and uracil; for the affinity-attenuated system generated by the previous method), SD-His/Leu plate (lacking methionine, lysine and uracil; for the PPI detection system generated by the new method) or SD-His plate (lacking methionine, lysine, uracil and leucine; for the affinity-enhanced system generated by the new method).

## Additional Information

**How to cite this article**: Kaishima, M. *et al.* Gγ recruitment systems specifically select PPI and affinity-enhanced candidate proteins that interact with membrane protein targets. *Sci. Rep.* **5**, 16723; doi: 10.1038/srep16723 (2015).

## Supplementary Material

Supplementary Information

## Figures and Tables

**Table 1:** Yeast strains used in this study.

Strain	Relevant feature	Source
BY4741	*MAT***a** *his3*Δ*1 ura3*Δ*0 leu2*Δ*0 met15*Δ	67
BY4742	*MAT*α *his3*Δ*1 ura3*Δ*0 leu2*Δ*0 lys2*Δ*0*	67
MC-F1	BY4741 *fig1*::*FIG1-EGFP*	43
MC-FC	MC-F1 *ste18*Δ::*kanMX4-P*_*PGK1*_*-Fc-Ste18C*	Present study
MC-FN	MC-F1 *ste18*Δ::*kanMX4-P*_*PGK1*_*-Gpa1N-Fc*	Present study
FC-GW	MC-F1 *ste18*Δ::*kanMX4-P*_*PGK1*_*-Fc-Ste18C his3*Δ::*URA3-P*_*STE18*_*-Gγ*_*cyto*_*-Z*_*WT*_	Present study
FC-GK	MC-F1 *ste18*Δ::*kanMX4-P*_*PGK1*_*-Fc-Ste18C his3*Δ::*URA3-P*_*STE18*_*-Gγ*_*cyto*_*-Z*_*K35A*_	Present study
FC-GI	MC-F1 *ste18*Δ::*kanMX4-P*_*PGK1*_*-Fc-Ste18C his3*Δ::*URA3-P*_*STE18*_*-Gγ*_*cyto*_*-Z*_*I31A*_	Present study
FC-G9	MC-F1 *ste18*Δ::*kanMX4-P*_*PGK1*_*-Fc-Ste18C his3*Δ::*URA3-P*_*STE18*_*-Gγ*_*cyto*_*-Z*_*955*_	Present study
FN-GW	MC-F1 *ste18*Δ::*kanMX4-P*_*PGK1*_*-Gpa1N-Fc his3*Δ::*URA3-P*_*STE18*_*-Gγ*_*cyto*_*-Z*_*WT*_	Present study
FN-GK	MC-F1 *ste18*Δ::*kanMX4-P*_*PGK1*_*-Gpa1N-Fc his3*Δ::*URA3-P*_*STE18*_*-Gγ*_*cyto*_*-Z*_*K35A*_	Present study
FN-GI	MC-F1 *ste18*Δ::*kanMX4-P*_*PGK1*_*-Gpa1N-Fc his3*Δ::*URA3-P*_*STE18*_*-Gγ*_*cyto*_*-Z*_*I31A*_	Present study
FN-G9	MC-F1 *ste18*Δ::*kanMX4-P*_*PGK1*_*-Gpa1N-Fc his3*Δ::*URA3-P*_*STE18*_*-Gγ*_*cyto*_*-Z*_*955*_	Present study
FC-W	MC-F1 *ste18*Δ::*kanMX4-P*_*PGK1*_*-Fc-Ste18C P*_*HOP2*_::*LEU2-P*_*PGK1*_*-Z*_*WT*_*-P*_*HOP2*_	Present study
FC-K	MC-F1 *ste18*Δ::*kanMX4-P*_*PGK1*_*-Fc-Ste18C P*_*HOP2*_::*LEU2-P*_*PGK1*_*-Z*_*K35A*_ *-P*_*HOP2*_	Present study
FC-I	MC-F1 *ste18*Δ::*kanMX4-P*_*PGK1*_*-Fc-Ste18C P*_*HOP2*_::*LEU2-P*_*PGK1*_*-Z*_*I31A*_*-P*_*HOP2*_	Present study
FC-9	MC-F1 *ste18*Δ::*kanMX4-P*_*PGK1*_*-Fc-Ste18C P*_*HOP2*_::*LEU2-P*_*PGK1*_*-Z*_*95*_*-P*_*HOP2*_	Present study
FN-W	MC-F1 *ste18*Δ::*kanMX4-P*_*PGK1*_*-Gpa1N-Fc P*_*HOP2*_::*LEU2-P*_*PGK1*_*-Z*_*WT*_*-P*_*HOP2*_	Present study
FN-K	MC-F1 *ste18*Δ::*kanMX4-P*_*PGK1*_*-Gpa1N-Fc P*_*HOP2*_::*LEU2-P*_*PGK1*_*-Z*_*K35A*_ *-P*_*HOP2*_	Present study
FN-I	MC-F1 *ste18*Δ::*kanMX4-P*_*PGK1*_*-Gpa1N-Fc P*_*HOP2*_::*LEU2-P*_*PGK1*_*-Z*_*I31A,cyto*_*-P*_*HOP2*_	Present study
FN-9	MC-F1 *ste18*Δ::*kanMX4-P*_*PGK1*_*-Gpa1N-Fc P*_*HOP2*_::*LEU2-P*_*PGK1*_*-Z*_*955,cyto*_*-P*_*HOP2*_	Present study
FC-GWW	MC-F1 *ste18*Δ::*kanMX4-P*_*PGK1*_*-Fc-Ste18C his3*Δ::*URA3-P*_*STE18*_*-Gγ*_*cyto*_*-Z*_*WT*_ *P*_*HOP2*_::*LEU2-P*_*PGK1*_*-Z*_*WT*_*-P*_*HOP2*_	Present study
FC-GWK	MC-F1 *ste18*Δ::*kanMX4-P*_*PGK1*_*-Fc-Ste18C his3*Δ::*URA3-P*_*STE18*_*-Gγ*_*cyto*_*-Z*_*WT*_ *P*_*HOP2*_::*LEU2-P*_*PGK1*_*-Z*_*K35A*_ *-P*_*HOP2*_	Present study
FC-GWI	MC-F1 *ste18*Δ::*kanMX4-P*_*PGK1*_*-Fc-Ste18C his3*Δ::*URA3-P*_*STE18*_*-Gγ*_*cyto*_*-Z*_*WT*_*P*_*HOP2*_::*LEU2-P*_*PGK1*_*-Z*_*I31A*_*-P*_*HOP2*_	Present study
FC-GW9	MC-F1 *ste18*Δ::*kanMX4-P*_*PGK1*_*-Fc-Ste18C his3*Δ::*URA3-P*_*STE18*_*-Gγ*_*cyto*_*-Z*_*WT*_*P*_*HOP2*_::*LEU2-P*_*PGK1*_*-Z*_*955*_*-P*_*HOP2*_	Present study
FC-GKW	MC-F1 *ste18*Δ::*kanMX4-P*_*PGK1*_*-Fc-Ste18C his3*Δ::*URA3-P*_*STE18*_*-Gγ*_*cyto*_*-Z*_*K35A*_ *P*_*HOP2*_::*LEU2-P*_*PGK1*_*-Z*_*WT*_*-P*_*HOP2*_	Present study
FC-GKK	MC-F1 *ste18*Δ::*kanMX4-P*_*PGK1*_*-Fc-Ste18C his3*Δ::*URA3-P*_*STE18*_*-Gγ*_*cyto*_*-Z*_*K35A*_ *P*_*HOP2*_::*LEU2-P*_*PGK1*_*-Z*_*K35A*_ *-P*_*HOP2*_	Present study
FC-GKI	MC-F1 *ste18*Δ::*kanMX4-P*_*PGK1*_*-Fc-Ste18C his3*Δ::*URA3-P*_*STE18*_*-Gγ*_*cyto*_*-Z*_*K35A*_ *P*_*HOP2*_::*LEU2-P*_*PGK1*_*-Z*_*I31A*_*-P*_*HOP2*_	Present study
FC-GK9	MC-F1 *ste18*Δ::*kanMX4-P*_*PGK1*_*-Fc-Ste18C his3*Δ::*URA3-P*_*STE18*_*-Gγ*_*cyto*_*-Z*_*K35A*_ *P*_*HOP2*_::*LEU2-P*_*PGK1*_*-Z*_*955*_*-P*_*HOP2*_	Present study
FC-GIW	MC-F1 *ste18*Δ::*kanMX4-P*_*PGK1*_*-Fc-Ste18C his3*Δ::*URA3-P*_*STE18*_*-Gγ*_*cyto*_*-Z*_*I31A*_ *P*_*HOP2*_::*LEU2-P*_*PGK1*_*-Z*_*WT*_*-P*_*HOP2*_	Present study
FC-GIK	MC-F1 *ste18*Δ::*kanMX4-P*_*PGK1*_*-Fc-Ste18C his3*Δ::*URA3-P*_*STE18*_*-Gγ*_*cyto*_*-Z*_*I31A*_ *P*_*HOP2*_::*LEU2-P*_*PGK1*_*-Z*_*K35A*_ *-P*_*HOP2*_	Present study
FC-GII	MC-F1 *ste18*Δ::*kanMX4-P*_*PGK1*_*-Fc-Ste18C his3*Δ::*URA3-P*_*STE18*_*-Gγ*_*cyto*_*-Z*_*I31A*_ *P*_*HOP2*_::*LEU2-P*_*PGK1*_*-Z*_*I31A*_*-P*_*HOP2*_	Present study
FC-GI9	MC-F1 *ste18*Δ::*kanMX4-P*_*PGK1*_*-Fc-Ste18C his3*Δ::*URA3-P*_*STE18*_*-Gγ*_*cyto*_*-Z*_*I31A*_ *P*_*HOP2*_::*LEU2-P*_*PGK1*_*-Z*_*955*_*-P*_*HOP2*_	Present study
FC-G9W	MC-F1 *ste18*Δ::*kanMX4-P*_*PGK1*_*-Fc-Ste18C his3*Δ::*URA3-P*_*STE18*_*-Gγ*_*cyto*_*-Z*_*955*_ *P*_*HOP2*_::*LEU2-P*_*PGK1*_*-Z*_*WT*_*-P*_*HOP2*_	Present study
FC-G9K	MC-F1 *ste18*Δ::*kanMX4-P*_*PGK1*_*-Fc-Ste18C his3*Δ::*URA3-P*_*STE18*_*-Gγ*_*cyto*_*-Z*_*955*_*P*_*HOP2*_::*LEU2-P*_*PGK1*_*-Z*_*K35A*_ *-P*_*HOP2*_	Present study
FC-G9I	MC-F1 *ste18*Δ::*kanMX4-P*_*PGK1*_*-Fc-Ste18C his3*Δ::*URA3-P*_*STE18*_*-Gγ*_*cyto*_*-Z*_*955*_ *P*_*HOP2*_::*LEU2-P*_*PGK1*_*-Z*_*I31A*_*-P*_*HOP2*_	Present study
FC-G99	MC-F1 *ste18*Δ::*kanMX4-P*_*PGK1*_*-Fc-Ste18C his3*Δ::*URA3-P*_*STE18*_*-Gγ*_*cyto*_*-Z*_*955*_ *P*_*HOP2*_::*LEU2-P*_*PGK1*_*-Z*_*955*_*-P*_*HOP2*_	Present study
FN-GWW	MC-F1 *ste18*Δ::*kanMX4-P*_*PGK1*_*-Gpa1N-Fc his3*Δ::*URA3-P*_*STE18*_*-Gγ*_*cyto*_*-Z*_*WT*_ *P*_*HOP2*_::*LEU2-P*_*PGK1*_*-Z*_*WT*_*-P*_*HOP2*_	Present study
FN-GWK	MC-F1 *ste18*Δ::*kanMX4-P*_*PGK1*_*-Gpa1N-Fc his3*Δ::*URA3-P*_*STE18*_*-Gγ*_*cyto*_*-Z*_*WT*_ *P*_*HOP2*_::*LEU2-P*_*PGK1*_*-Z*_*K35A*_ *-P*_*HOP2*_	Present study
FN-GWI	MC-F1 *ste18*Δ::*kanMX4-P*_*PGK1*_*-Gpa1N-Fc his3*Δ::*URA3-P*_*STE18*_*-Gγ*_*cyto*_*-Z*_*WT*_ *P*_*HOP2*_::*LEU2-P*_*PGK1*_*-Z*_*I31A*_*-P*_*HOP2*_	Present study
FN-GW9	MC-F1 *ste18*Δ::*kanMX4-P*_*PGK1*_*-Gpa1N-Fc his3*Δ::*URA3-P*_*STE18*_*-Gγ*_*cyto*_*-Z*_*WT*_ *P*_*HOP2*_::*LEU2-P*_*PGK1*_*-Z*_*955*_*-P*_*HOP2*_	Present study
FN-GKW	MC-F1 *ste18*Δ::*kanMX4-P*_*PGK1*_*-Gpa1N-Fc his3*Δ::*URA3-P*_*STE18*_*-Gγ*_*cyto*_*-Z*_*K35A*_ *P*_*HOP2*_::*LEU2-P*_*PGK1*_*-Z*_*WT*_*-P*_*HOP2*_	Present study
FN-GKK	MC-F1 *ste18*Δ::*kanMX4-P*_*PGK1*_*-Gpa1N-Fc his3*Δ::*URA3-P*_*STE18*_*-Gγ*_*cyto*_*-Z*_*K35A*_ *P*_*HOP2*_::*LEU2-P*_*PGK1*_*-Z*_*K35A*_ *-P*_*HOP2*_	Present study
FN-GKI	MC-F1 *ste18*Δ::*kanMX4-P*_*PGK1*_*-Gpa1N-Fc his3*Δ::*URA3-P*_*STE18*_*-Gγ*_*cyto*_*-Z*_*K35A*_ *P*_*HOP2*_::*LEU2-P*_*PGK1*_*-Z*_*I31A*_*-P*_*HOP2*_	Present study
FN-GK9	MC-F1 *ste18*Δ::*kanMX4-P*_*PGK1*_*-Gpa1N-Fc his3*Δ::*URA3-P*_*STE18*_*-Gγ*_*cyto*_*-Z*_*K35A*_ *P*_*HOP2*_::*LEU2-P*_*PGK1*_*-Z*_*955*_*-P*_*HOP2*_	Present study
FN-GIW	MC-F1 *ste18*Δ::*kanMX4-P*_*PGK1*_*-Gpa1N-Fc his3*Δ::*URA3-P*_*STE18*_*-Gγ*_*cyto*_*-Z*_*I31A*_ *P*_*HOP2*_::*LEU2-P*_*PGK1*_*-Z*_*WT*_*-P*_*HOP2*_	Present study
FN-GIK	MC-F1 *ste18*Δ::*kanMX4-P*_*PGK1*_*-Gpa1N-Fc his3*Δ::*URA3-P*_*STE18*_*-Gγ*_*cyto*_*-Z*_*I31A*_ *P*_*HOP2*_::*LEU2-P*_*PGK1*_*-Z*_*K35A*_ *-P*_*HOP2*_	Present study
FN-GII	MC-F1 *ste18*Δ::*kanMX4-P*_*PGK1*_*-Gpa1N-Fc his3*Δ::*URA3-P*_*STE18*_*-Gγ*_*cyto*_*-Z*_*I31A*_ *P*_*HOP2*_::*LEU2-P*_*PGK1*_*-Z*_*I31A*_*-P*_*HOP2*_	Present study
FN-GI9	MC-F1 *ste18*Δ::*kanMX4-P*_*PGK1*_*-Gpa1N-Fc his3*Δ::*URA3-P*_*STE18*_*-Gγ*_*cyto*_*-Z*_*I31A*_ *P*_*HOP2*_::*LEU2-P*_*PGK1*_*-Z*_*955*_*-P*_*HOP2*_	Present study
FN-G9W	MC-F1 *ste18*Δ::*kanMX4-P*_*PGK1*_*-Gpa1N-Fc his3*Δ::*URA3-P*_*STE18*_*-Gγ*_*cyto*_*-Z*_*955*_ *P*_*HOP2*_::*LEU2-P*_*PGK1*_*-Z*_*WT*_*-P*_*HOP2*_	Present study
FN-G9K	MC-F1 *ste18*Δ::*kanMX4-P*_*PGK1*_*-Gpa1N-Fc his3*Δ::*URA3-P*_*STE18*_*-Gγ*_*cyto*_*-Z*_*955*_ *P*_*HOP2*_::*LEU2-P*_*PGK1*_*-Z*_*K35A*_ *-P*_*HOP2*_	Present study
FN-G9I	MC-F1 *ste18*Δ::*kanMX4-P*_*PGK1*_*-Gpa1N-Fc his3*Δ::*URA3-P*_*STE18*_*-Gγ*_*cyto*_*-Z*_*955*_ *P*_*HOP2*_::*LEU2-P*_*PGK1*_*-Z*_*I31A*_*-P*_*HOP2*_	Present study
FN-G99	MC-F1 *ste18*Δ::*kanMX4-P*_*PGK1*_*-Gpa1N-Fc his3*Δ::*URA3-P*_*STE18*_*-Gγ*_*cyto*_*-Z*_*955*_ *P*_*HOP2*_::*LEU2-P*_*PGK1*_*-Z*_*955*_*-P*_*HOP2*_	Present study
MC-ErC	MC-F1 *ste18*Δ::*kanMX4-P*_*PGK1*_*-EGFR*_*L834R,cyto*_*-Ras1C*	Present study
MC-EsC	MC-F1 *ste18*Δ::*kanMX4-P*_*PGK1*_*-EGFR*_*L834R,cyto*_*-Ste18C*	Present study
MC-EgN	MC-F1 *ste18*Δ::*kanMX4-P*_*PGK1*_*-Gpa1N-EGFR*_*L834R,cyto*_	Present study
ErC-grbG	MC-F1 *ste18*Δ::*kanMX4-P*_*PGK1*_*-EGFR*_*L834R,cyto*_*-Ras1C his3*Δ::*URA3-P*_*STE18*_*-Grb2-Gγ*_*cyto*_	Present study
EsC-grbG	MC-F1 *ste18*Δ::*kanMX4-P*_*PGK1*_*-EGFR*_*L834R,cyto*_*-Ste18C his3*Δ::*URA3-P*_*STE18*_*-Grb2-Gγ*_*cyto*_	Present study
EgN-grbG	MC-F1 *ste18*Δ::*kanMX4-P*_*PGK1*_*-Gpa1N-EGFR*_*L834R,cyto*_ *his3*Δ::*URA3-P*_*STE18*_*-Grb2-Gγ*_*cyto*_	Present study
ErC-Ggrb	MC-F1 *ste18*Δ::*kanMX4-P*_*PGK1*_*-EGFR*_*L834R,cyto*_*-Ras1C his3*Δ::*URA3-P*_*STE18*_ *-Gγ*_*cyto*_*-Grb2*	Present study
EsC-Ggrb	MC-F1 *ste18*Δ::*kanMX4-P*_*PGK1*_*-EGFR*_*L834R,cyto*_*-Ste18C his3*Δ::*URA3-P*_*STE18*_ *-Gγ*_*cyto*_*-Grb2*	Present study
EgN-Ggrb	MC-F1 *ste18*Δ::*kanMX4-P*_*PGK1*_*-Gpa1N-EGFR*_*L834R,cyto*_ *his3*Δ::*URA3-P*_*STE18*_ *-Gγ*_*cyto*_*-Grb2*	Present study
ErC-grbG-E89K	MC-F1 *ste18*Δ::*kanMX4-P_PGK1_-EGFR_L834R,cyto_-Ras1C his3*Δ::*URA3-P_STE18_-Grb2-Gγ_cyto_ PHOP2*::*LEU2-P_PGK1_-Grb2_E89K_ -P_HOP2P_*	Present study
ErC-grbG-R86G	MC-F1 *ste18*Δ::*kanMX4-P_PGK1_-EGFR_L834R,cyto_-Ras1C his3*Δ::*URA3-P_STE18_-Grb2-Gγ_cyto_ P_HOP2P_*::*LEU2-P_PGK1_-Grb2_R86G_-P_HOP2P_*	Present study
ErC-grbG-LEU	MC-F1 *ste18*Δ::*kanMX4-P_PGK1_-EGFR_L834R,cyto_-Ras1C his3*Δ::*URA3-P_STE18_-Grb2-Gγ_cyto_ P_HOP2P_*::*LEU2-P_HOP2P_*	Present study

**Table 2:** List of plasmids used in this study.

Plasmids	Genotype	Reference
pGK425	Expression vector containing *PGK1* promoter, 2*μ* origin and *LEU2* marker	68
pGK425-Gpa1N	N-terminus of Gpa1 (9 a.a.) expression, in pGK425	This study
pGK425-Ste18C	C-terminus of Ste18 (9 a.a.) expression, in pGK425	This study
pGK425-Ras1C	C-terminus of Ras1 (10 a.a.) expression, in pGK425	This study
pGK425-Gpa1N-Fc	Fc protein expression, in pGK425-Gpa1N	This study
pGK425-Fc-Ste18C	Fc protein expression, in pGK425-Ste18C	This study
pGK426-GPTK	*URA3-STE18 promoter-kanMX4-STE18 terminator* in pGK426	42
pUMGPTK-Gpa1N-Fc	*URA3-STE18p-PGK1 promoter -Gpa1N (9 a.a.)-Fc- PGK1 terminator -kanMX4-STE18t* in pGK426-GPTK	This study
pUMGPTK-Fc-Ste18C	*URA3-STE18p-PGK1 promoter -Fc-Ste18C (9 a.a.)- PGK1 terminator -kanMX4-STE18t* in pGK426-GPTK	This study
pGK413	Expression vector containing *PGK1* promoter, *CEN/ARS* single-copy origin and *HIS3* marker	68
pGK413-Gγ-EZWT	Gγ_cyto_-Z_WT_ fusion expression, in pGK413	This study
pGK413-Gγ-EZK35A	Gγ_cyto_-Z_K35A_ fusion expression, in pGK413	This study
pGK413-Gγ-EZI31A	Gγ_cyto_ -Z_I31A_ fusion expression, in pGK413	This study
pGK413-Gγ-EZ955	Gγ_cyto_ -Z_955_ fusion expression, in pGK413	This study
pUSTE18p-Gγcyto	*URA3-STE18 promoter-Gγ*_*cyto*_*-PGK1 terminator* in pGK426	This study
pUSTE18p-Gγcyto-HIS3t	*URA3-STE18 promoter-Gγ*_*cyto*_*-PGK1 terminator-HIS3 terminator* in pGK426	This study
pUSTE18p-Gγcyto-ZWT-H	*URA3-STE18 promoter-*Gγ_cyto_-Z_WT_*-PGK1 terminator* in pUSTE18p-Gγcyto-HIS3t	This study
pUSTE18p-Gγcyto-ZK35A-H	*URA3-STE18 promoter-Gγ*_*cyto*_*-Z*_*K35A*_*-PGK1 terminator* in pUSTE18p-Gγcyto-HIS3t	This study
pUSTE18p-Gγcyto-ZI31A-H	*URA3-STE18 promoter-Gγ*_*cyto*_*-Z*_*I31A*_*-PGK1 terminator* in pUSTE18p-Gγcyto-HIS3t	This study
pUSTE18p-Gγcyto-Z955-H	*URA3-STE18 promoter-Gγ*_*cyto*_*-Z*_*955*_*-PGK1 terminator* in pUSTE18p-Gγcyto-HIS3t	This study
pGK415	Expression vector containing *PGK1* promoter, *CEN/ARS* single-copy origin and *LEU2* marker	68
pGK-LsZWTc	Z_WT_ expression, in pGK415	51
pGK-LsZK35Ac	Z_K35A_ expression, in pGK415	41
pGK-LsZI31Ac	Z_I31A_ expression, in pGK415	41
pGK-LsZ955c	Z_955_ expression, in pGK415	41
pGK-LsZWTc-HOP2p	*LEU2-PGK promoter -Z*_*WT*_*-PGK terminator -HOP2 promoter* in pGK415	This study
pGK-LsZK35Ac-HOP2p	*LEU2-PGK promoter -Z*_*K35A*_*-PGK terminator -HOP2 promoter* in pGK415	This study
pGK-LsZI31Ac-HOP2p	*LEU2-PGK promoter -Z*_*I31A*_*-PGK terminator -HOP2 promoter* in pGK415	This study
pGK-LsZ955c-HOP2p	*LEU2-PGK promoter -Z*_*955*_*-PGK terminator -HOP2 promoter* in pGK415	This study
pGK425-Gpa1N-EGFR(LR)	EGFR_L834R,cyto_ expression, in pGK425-Gpa1N	This study
pGK425-EGFR(LR)-Ste18C	EGFR_L834R,cyto_ expression, in pGK425-Ste18C	This study
pGK425-EGFR(LR)-Ste18C	EGFR_L834R,cyto_ expression, in pGK425-Ste18C	This study
pUMGPTK-Gpa1N-EGFR(LR)	*URA3-STE18p-PGK1 promote -Gpa1N (9 a.a.)-EGFR*_*L834R,cyto*_*-PGK1 terminator -kanMX4-STE18t* in pGK426-GPTK	This study
pUMGPTK-EGFR(LR)-Ste18C	*URA3-STE18p-PGK1 promoter-EGFR*_*L834R,cyto*_*-Ste18C (9 a.a.)-PGK1 terminator -kanMX4-STE18t* in pGK426-GPTK	This study
pUMGPTK-EGFR(LR)-Ras1C	*URA3-STE18p-PGK1 promoter -EGFR*_*L834R,cyto*_*-Ras1C (10 a.a.)-PGK1 terminator -kanMX4-STE18t in pGK426-G*PTK	This study
pGK413-Grb2-Gγ	Grb2-Gγ_cyto_ fusion expression, in pGK413	This study
pGK416	Expression vector containing *PGK1* promoter, *CEN/ARS* single-copy origin and *URA3* marker	68
Ste18p-416	*URA3-STE18 promoter-PGK1 terminator* in pGK416	This study
pUSTE18p-c-Gγcyto	*URA3-STE18 promoter-Gγ*_*cyto*_*(w/ stop codon)-PGK1 terminator* in pGK416	This study
pUSTE18p-c-Gγcyto-HIS3t	*URA3-STE18 promoter-Gγ*_*cyto*_*(w/ stop codon)-PGK1 terminator-HIS3 terminator* in pGK416	This study
pUSTE18p-Grb2-Gγcyto-HIS3t	*URA3-STE18 promoter-Grb2-Gγ*_*cyto*_*-PGK1 terminator* in pUSTE18p-c-Gγcyto-HIS3t	This study
pUSTE18p-Gγcyto-Grb2-HIS3t	*URA3-STE18 promoter-Gγ*_*cyto*_*-Grb2-PGK1 terminator* in pUSTE18p-Gγcyto-HIS3t	This study
pUSTE18p-Grb2(R86G)-Gγcyto-HIS3t	*URA3-STE18 promoter-Grb2*_*R86G*_*-Gγ*_*cyto*_*-PGK1 terminator* in pUSTE18p-Gγcyto-HIS3t	This study
pUSTE18p-Grb2(E89K)-Gγcyto-HIS3t	*URA3-STE18 promoter-Grb2*_*E89K*_*-Gγ*_*cyto*_*-PGK1 terminator* in pUSTE18p-Gγcyto-HIS3t	This study
pGK415-HOP2p	*URA3-PGK1 promoter-PGK1 terminator-HOP2 promoter* in pGK415	This study
pGK-LsGrb2-HOP	*LEU2-PGK1 promoter-Grb2-PGK1 terminator-HOP2 promoter* in pGK415	This study
pGK-LsGrb2(R86G)-HOP	*LEU2-PGK1 promoter-Grb2*_*R86G*_*-PGK1 terminator-HOP2 promoter* in pGK415	This study
pGK-LsGrb2(E89K)-HOP	*LEU2-PGK1 promoter-Grb2*_*E89K*_*-PGK1 terminator-HOP2 promoter* in pGK415	This study
