# Supplementary material for: Gγ recruitment systems specifically select PPI and affinity-enhanced candidate proteins that interact with membrane protein targets
Source: Sci Rep. 2015 Nov 19;5:16723. doi: 10.1038/srep16723 (PMC4652169; doi:10.1038/srep16723)
Supplement: Supplementary Information [file srep16723-s1.pdf]

Supplementary Information

Gγ recruitment systems specifically select PPI and affinity-enhanced candidate proteins that interact with membrane protein targets

Misato Kaishima[1], Jun Ishii[2], Nobuo Fukuda[3] and Akihiko Kondo[1]

Table S1. List of primers used in this study.

No.	Primer Name	Sequence (5' to 3')
1	XhoI-PGK5'-fw	aaaactcgagaaagatgccgatttgggc
2	PGK5-Gpa1N-MCS-rv	ggactagtccatggcttaaggtcgactattgtttgcgtactcactgtacaccccatgctagcgttttatatttgttgtaa
3	MCS(Gpa1N)-rv	ttttagatctacgcgtggatccgaattctctagacccgggactagtccatggcttaagg
4	PGK5-Ste18C-MCS-rv	tgaacgcgtggatccgaattctctagacccgggactagtccatggcttaaggtcgacgctagcgttttatatttgttgta
5	MCS(Ste18C)-rv	ttttagatctttacataagcgtacaacaaacactatttgaacgcgtggatccgaattc
6	PGK5-Ras1C-MCS-rv	ttcacgcgtggatccgaattctctagacccgggactagtccatggcttaaggtcgacgctagcgttttatatttgttgta
7	MCS(Ras1C)-rv	ttttagatctttaacaaattatacaacaaccaccactagattcacgcgtggatccgaattc
8	SalI-Fc(1-16)-fw	aaaagtcgacgggggaccgtcagtct
9	BamHI-end-Fc-rv	ttttggatcctcatttacccggagacaggg
10	SalI-start-Fc(1-16)-fw	aaaagtcgacatgggggaccgtcagtct
11	BamHI-Fc-rv	ttttggatcctttacccggagacagggaga
12	XhoI-PGK5-fw (In-Fusion)	aattctaagactcgagaaagatgccgatttgggc
13	XhoI-PGK3-rv (In Fusion)	aaacagatctctcgagagctttaacgaacgc
14	SalI-start-Ste18mu-fw	aaaagtcgacatgacatcagtt
15	E domain-Flag-Ste18mu-rv	ggcttcatcgtgttgcgccttgtcatcgtcatccttgtagtcaacactatttgagtt
16	E domain-Z(1-5)-fw	gcgcaacacgatgaagccgtaga
17	EcoRI-end-Z(160-174)-rv	tttttgaattcttatttcggcgcctgagc
18	XhoI-Ste18p-fw	aaaactcgagatattatatatatatatatagggtcgtatatatatcgtgcgtcttcttcctt
19	NheI-Ste18m-rv	ttttgctagcaacactatttgagtttgacatttggctattttttgagcatttttcaaac
20	NotI-HIS3t-fw	aaaagcggccgctgacaccgattatttaaagctgcagcatacgatatatatacatgtgtata
21	SacI-HIS3t-rv	ttttgagctcggagccataatgacagcagttgggtaggcctttctttggtaaaaggagcc
22	SalI-EZ(1-15)-fw	aaaagtcgacgcgcaacacgatgaagccgtagacaacaaattc
23	BamHI-end-Z(160-174)-rv	ttttggatccttatttcggcgcctgagc
24	NotI-Hop2p-fw	aaaagcggccgcttaaagcaagggtaa
25	SacI-Hop2p-rv	ttttgagctcatctttcaaatagag
26	SalI-EGFR(LR)-fw	aatagtcgacagtggagaagctcccaaccaagctc
27	MluI-end-EGFR(LR)-rv	atctacgcgtttatgctccaataaattcactgctt
28	SalI-start-EGFR(LR)-fw	tagcgtcgacatgagtggagaagctcccaaccaag
29	MluI-EGFR(LR)-rv	catcacgcgttgctccaataaattcactgctttgt
30	SalI-start-Grb2-fw(IF)	atataaaacgctagcgtcgacatggaagccatcgccaaa
31	EcoRI-end-Ste18m-rv(IF)	tttatttcagatctgaattcttaaacactatttgagttt
32	XhoI-Ste18p-fw	aaaactcgagatattatatatatatatatagggtcgtatatatatcgtgcgtcttcttcctt
33	NheI-Ste18p-rv	ttttgctagctcttagaattattga
34	XbaI-Ste18m-fw	cgggtctagaacatcagttcaaaactct

35	EcoRI-end-Ste18m-rv	atctgaattcttaaacactatttgagtttg
36	NheI-start-Grb2-fw	aaaagctagcatggaagccatcgc
37	XmaI-Grb2-rv	tagacccggggacgttccggttcacgggggtgaca
38	NheI-Grb2-fw	tgttgctagcgaagccatcgccaaatatgacttca
39	XmaI-end-Grb2-rv	tagacccgggttagacgttccggttcacgggggtg
40	SalI-start-Grb2-fw (IF)	atataaaacgctagcgtcgacatggaagccatcgccaaa
41	XmaI-end-Grb2-rv (IF)	tctgaattctctagacccgggttagacgttccggttcac
42	Grb2 R86G-rv	ctctcactctcaccgataagaaaggc
43	Grb2 R86G-fw	gcctttcttatcggtgagagtgagag
44	Grb2 E89K-rv	cccaggagcgcttttactctctcggataag
45	Grb2 E89K-fw	cttatccgagagagtaaaagcgctcctggg
46	Ste18pro-fw	atattatatatatatatagggtcgt
47	Ste18t-rv	aaattatagaaagcagtagataaaa
48	HIS3pro80-URA3-fw	tatataaagtaatgtgatttcttcgaagaatatactaaaaaatgagcaggcaagataaacgaaggcaaagttcaattcatcatttttttt ttattctttt
49	HIS3t end40-rv	ggagccataatgacagcagttgggtaggcctttctttggt
50	Hop2p 150-200-LEU2-fw	atacaattaattgacatcagcagacagcaaatgcacttgatatacgcagctcgactacgtcgtaaggccgt
51	Hop2p 800-rv	atctttcaaatagagcctgg

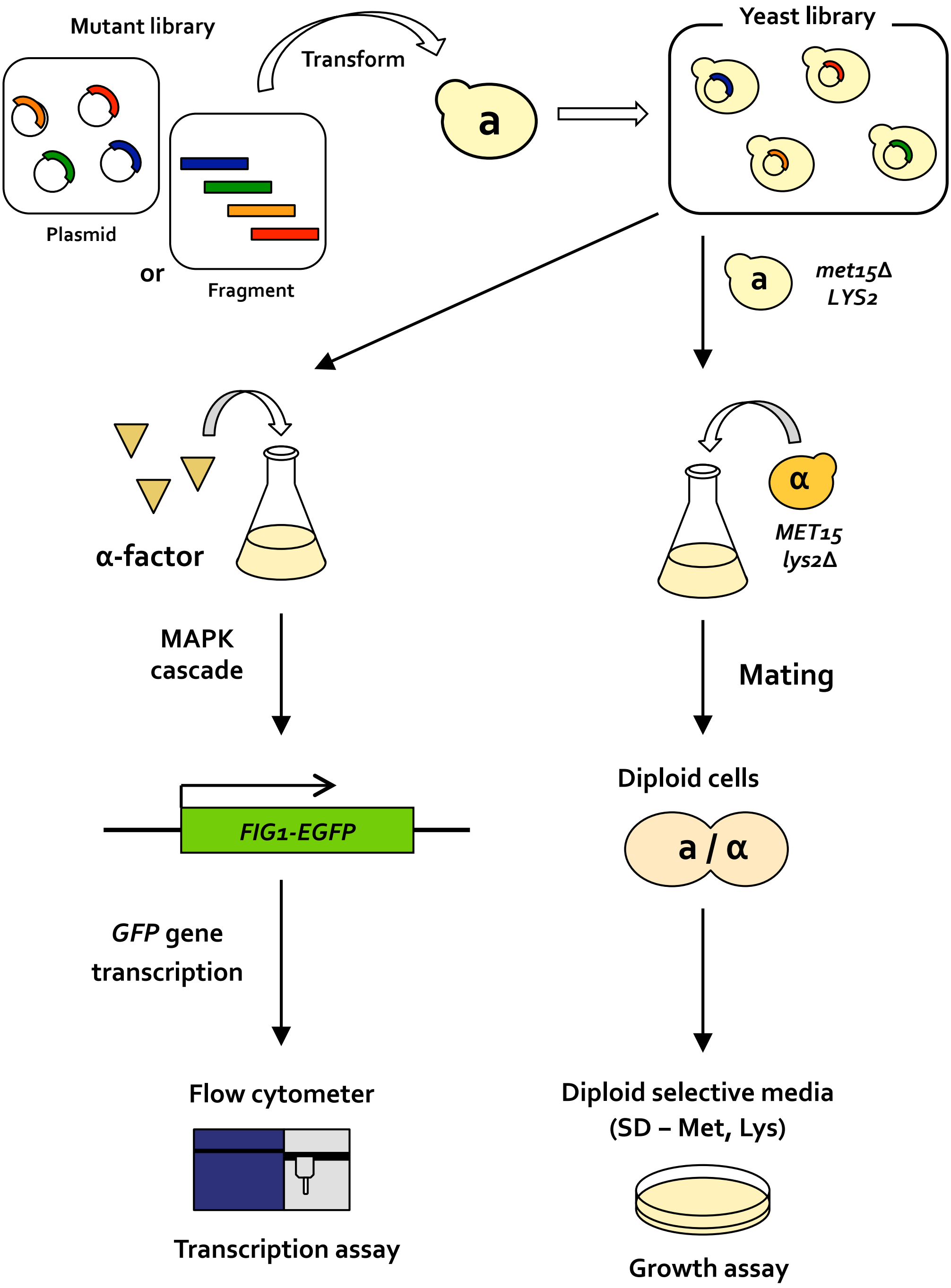

Figure S1. Flow diagram of the screening procedure for the competitive Gγ recruitment system for target membrane proteins.

Two selection methods are available to screen affinity-altered proteins. One method is to use the *GFP* reporter gene. When target candidate proteins are expressed in the yeast cells and interact with each other, they induce *GFP* expression and are isolated by flow cytometry. Another method is to use yeast mating detected using a growth assay. When target candidate proteins are expressed in yeast cells, they restore mating ability and grow on the diploid-selective medium.

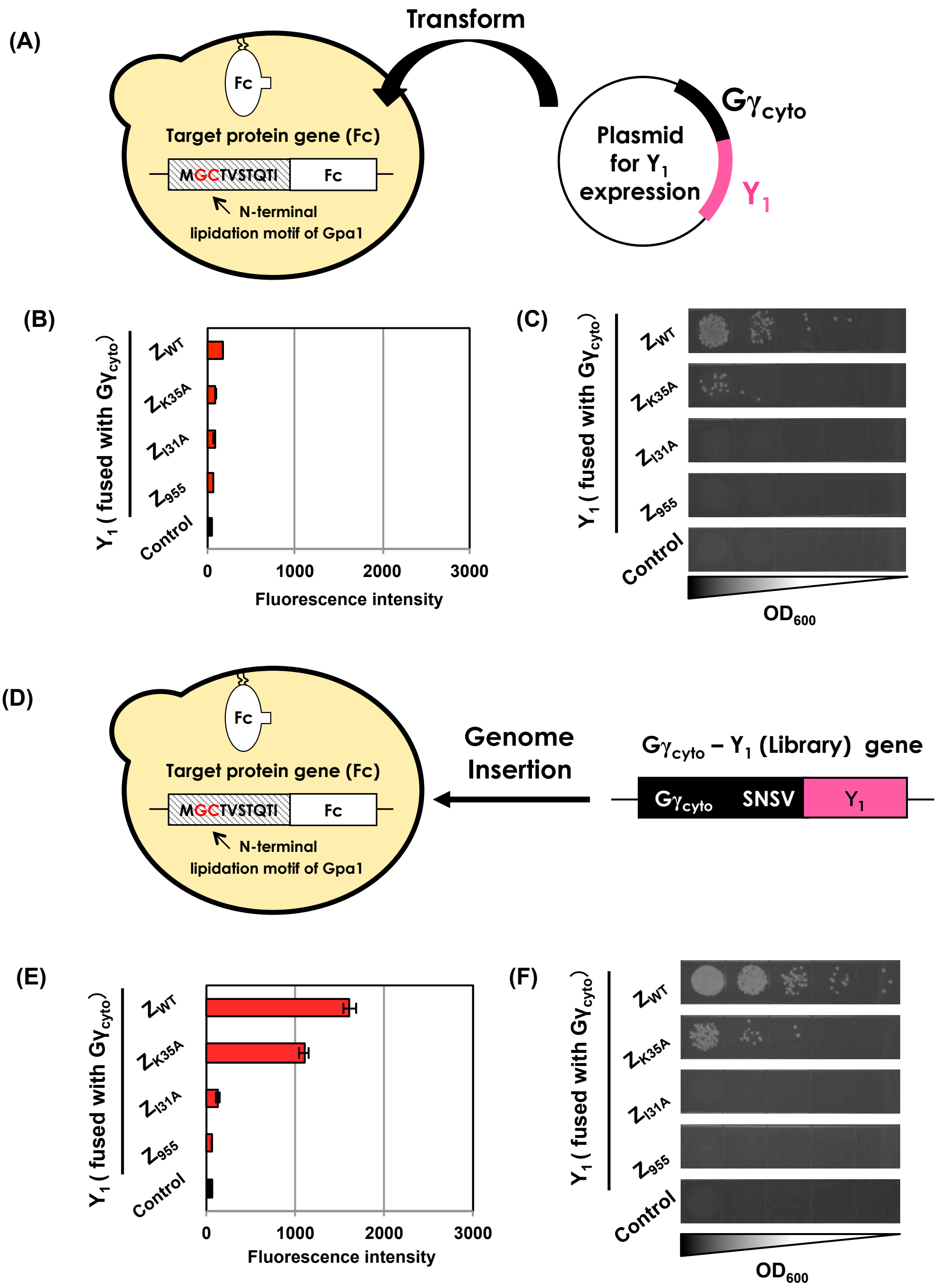

Figure S2. Selection of Z variants binding to membrane-associated target Fc proteins using previous and new methods for Gγ recruitment systems.

(A) Previous recipe of the Gγ recruitment system for membrane proteins as targets. (B and C) The flow cytometry analyses and mating growth assay. Fluorescence and growth intensities of the engineered strains expressing N-terminally membrane-associated Fc via stable integrating into the yeast chromosome as well as cytosolic Z variants fused to $G\gamma_{cyto}$ 'Y_1' via autonomous replication plasmids. The control yeast shows the strain without the expression of 'Y_1' fused to $G\gamma_{cyto}$ (transformed with pGK413 mock vector). (D) New recipe of Gγ recruitment system for membrane proteins as target. (E and F) Flow cytometric analyses and mating growth assay. The fluorescence and growth intensities of the engineered strains expressing N-terminally membrane-associated Fc and cytosolic Z variants fused to $G\gamma_{cyto}$ via stable integration into the yeast chromosome. The control yeast shows the strain without the expression of 'Y_1' fused to $G\gamma_{cyto}$ (MC-FN in Table 1).

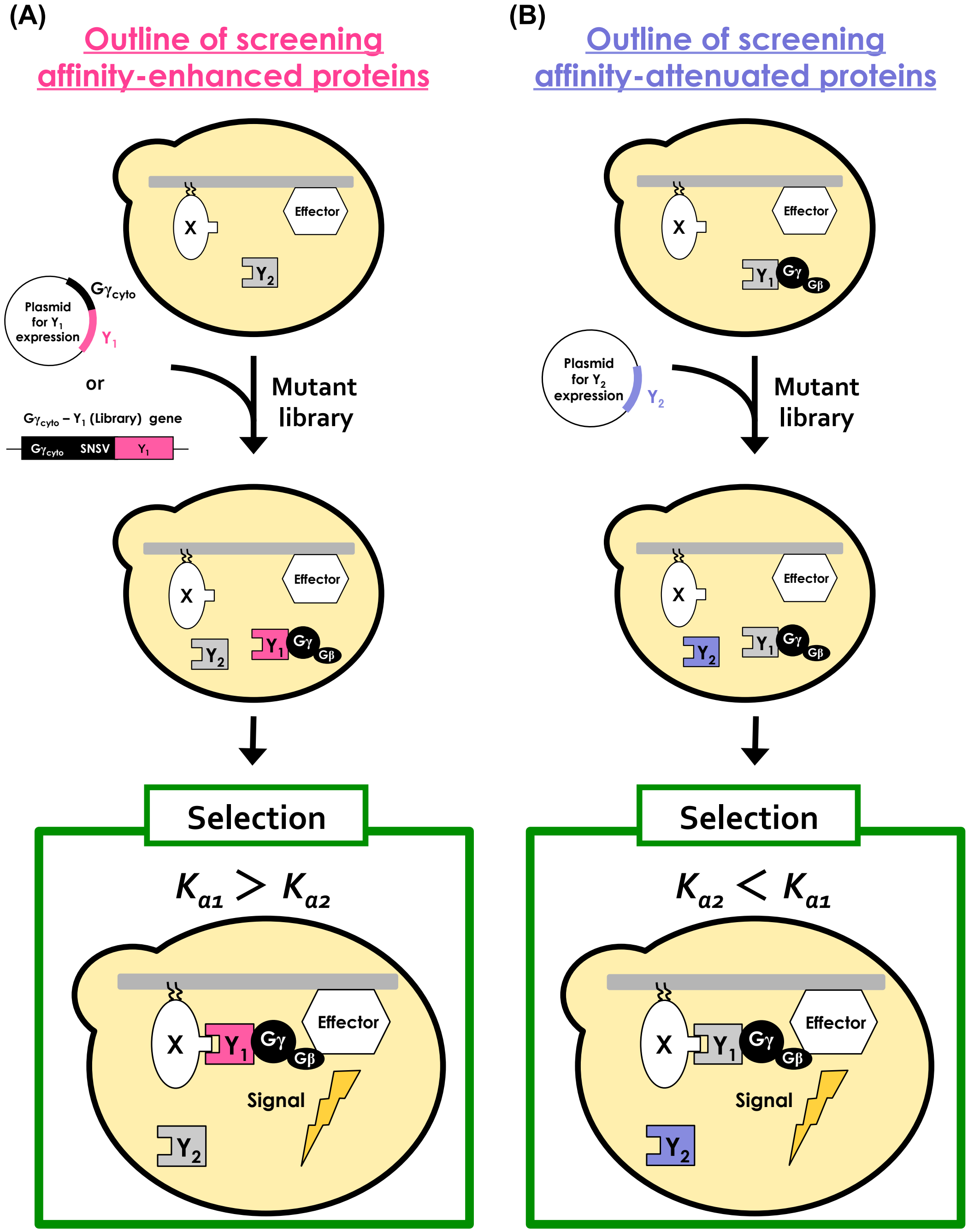

Figure S3. Flow diagram of the selection principle behind the competitive Gγ recruitment system for target membrane proteins.
(A) Outline for the selection of desirable affinity-enhanced proteins. The yeast strain expressing target membrane protein 'X' and the competitor protein 'Y_2' in the cytosol is transformed with the plasmid or integrated to the DNA cassettes expressing the mutant library fused with the Gγ mutant ($G\gamma_{cyto}$-Y_1) in the cytosol. Preferential binding of "X" to "Y_1" ($K_{a1}>K_{a2}$) restores the signaling function and permits the selective screening of affinity-enhanced proteins. (B) Outline for screening affinity-attenuated proteins. The yeast strain expressing target membrane protein 'X' and a competitor protein 'Y_1' fused with the Gγ mutant ($G\gamma_{cyto}$-Y_1) is transformed with the plasmid or integrated to the DNA cassettes expressing the mutant library in the cytosol (Y_2). Preferential binding of "X" to "Y_1" ($K_{a2}<K_{a1}$) restores the signaling function and permits the selective screening of affinity-attenuated proteins.

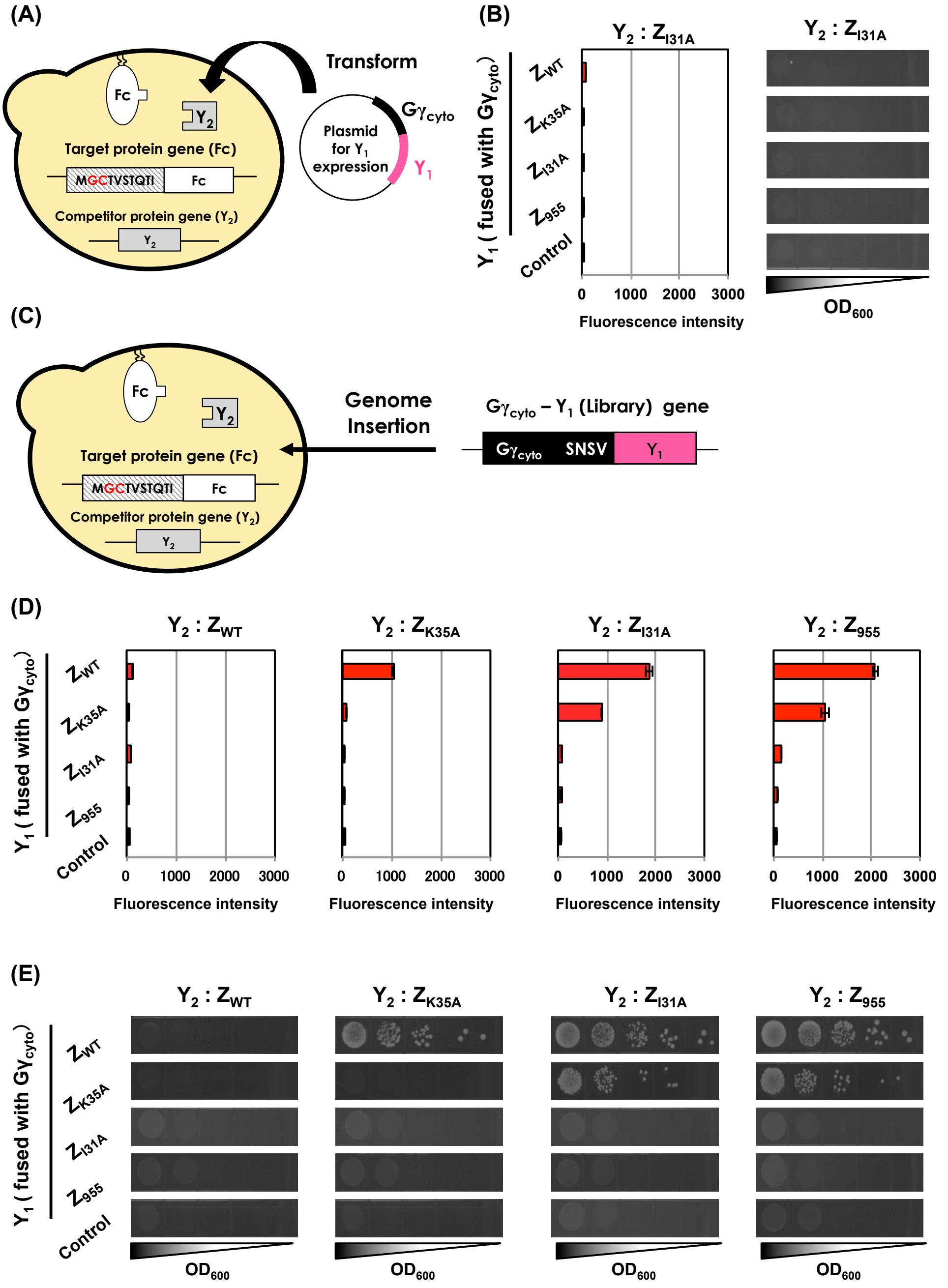

Figure. S4 Competitive selection of Z variants with higher affinities for membrane-associated target Fc using previous and new methods for affinity-enhanced systems.

(A) Previous recipe of the affinity-enhanced system for membrane proteins as targets. (B) Flow cytometry analyses and mating growth assay. The fluorescence and growth intensities of the engineered strains expressing N-terminally membrane-associated Fc and competitor Z_{I31A} as cytosolic 'Y_2' via stable integration into the yeast chromosome as well as cytosolic Z variants 'Y_1' fused to $G\gamma_{cyto}$ by the autonomous replication plasmids. Control yeast strains lacked the expression of 'Y_1' fused to $G\gamma_{cyto}$ (transformed with pGK413 mock vector). (C) New recipe of affinity-enhanced system for membrane proteins as targets. (D and E) Flow cytometry analyses and mating growth assay. The fluorescence and growth intensities of the engineered strains expressing N-terminally membrane-associated Fc and cytosolic Z variants fused to $G\gamma_{cyto}$, competitor cytosolic Z variants 'Y_2' and cytosolic Z variants 'Y_1' fused to $G\gamma_{cyto}$ via stable integration into the yeast chromosome. The control yeast shows the strain without the expression of 'Y_1' fused to $G\gamma_{cyto}$.

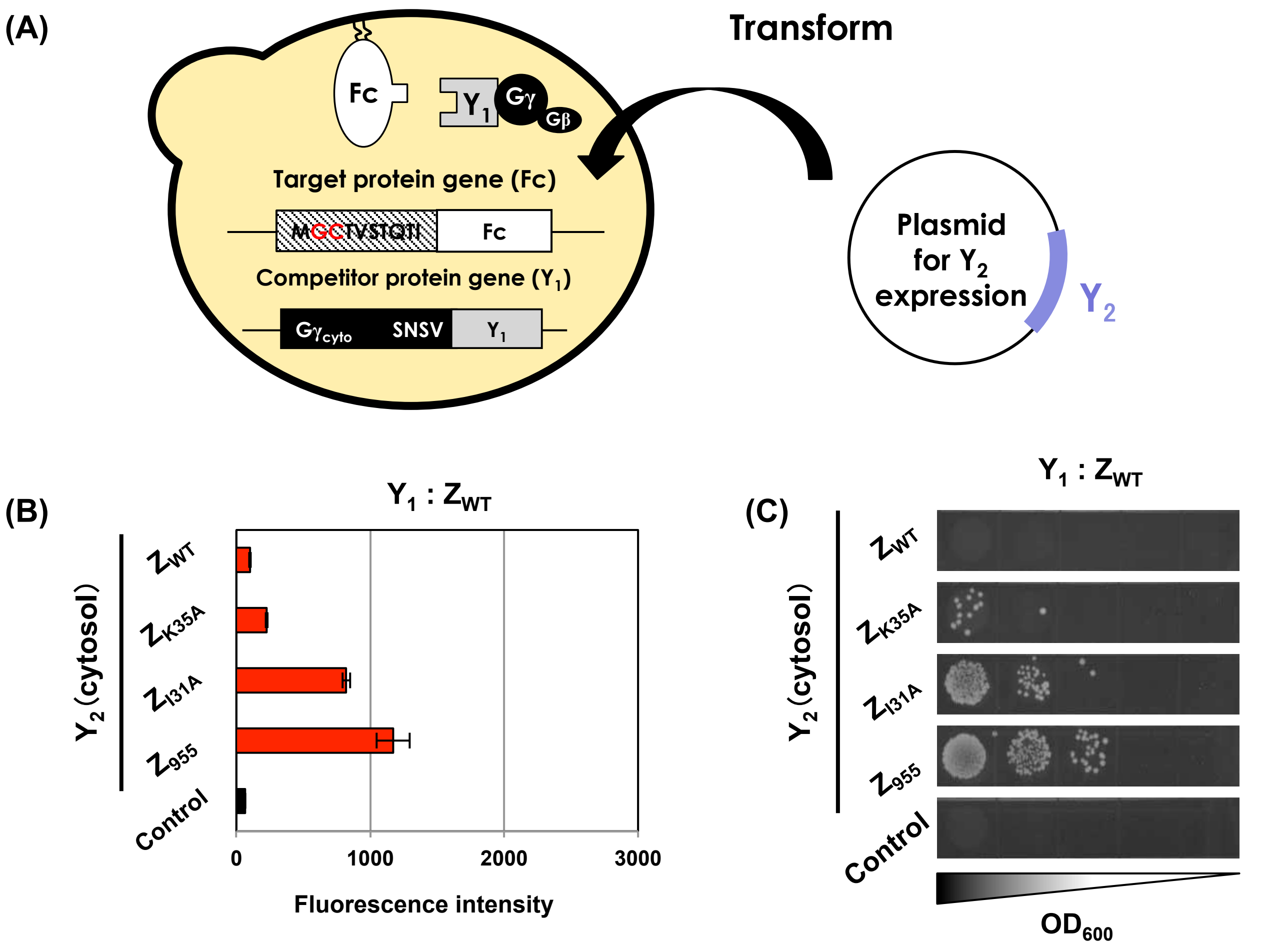

Figure. S5 Competitive selection of Z variants with lower affinities for membrane-associated target Fc using the previous method for affinity-attenuated system.

(A) Previous recipe of the affinity-attenuated system for membrane proteins as targets. (B, C) Flow cytometry analyses and mating growth assay. The fluorescence and growth intensities of the engineered strains expressing N-terminally membrane-associated Fc and competitor Z_{WT} as cytosolic 'Y_1' fused to $G\gamma_{cyto}$ via stable integration into the yeast chromosome and cytosolic Z variants 'Y_2' viaautonomous replication plasmids. Control yeast shows the strain without the expression of 'Y_1' fused to $G\gamma_{cyto}$ and cytosolic Z variants 'Y_2.'

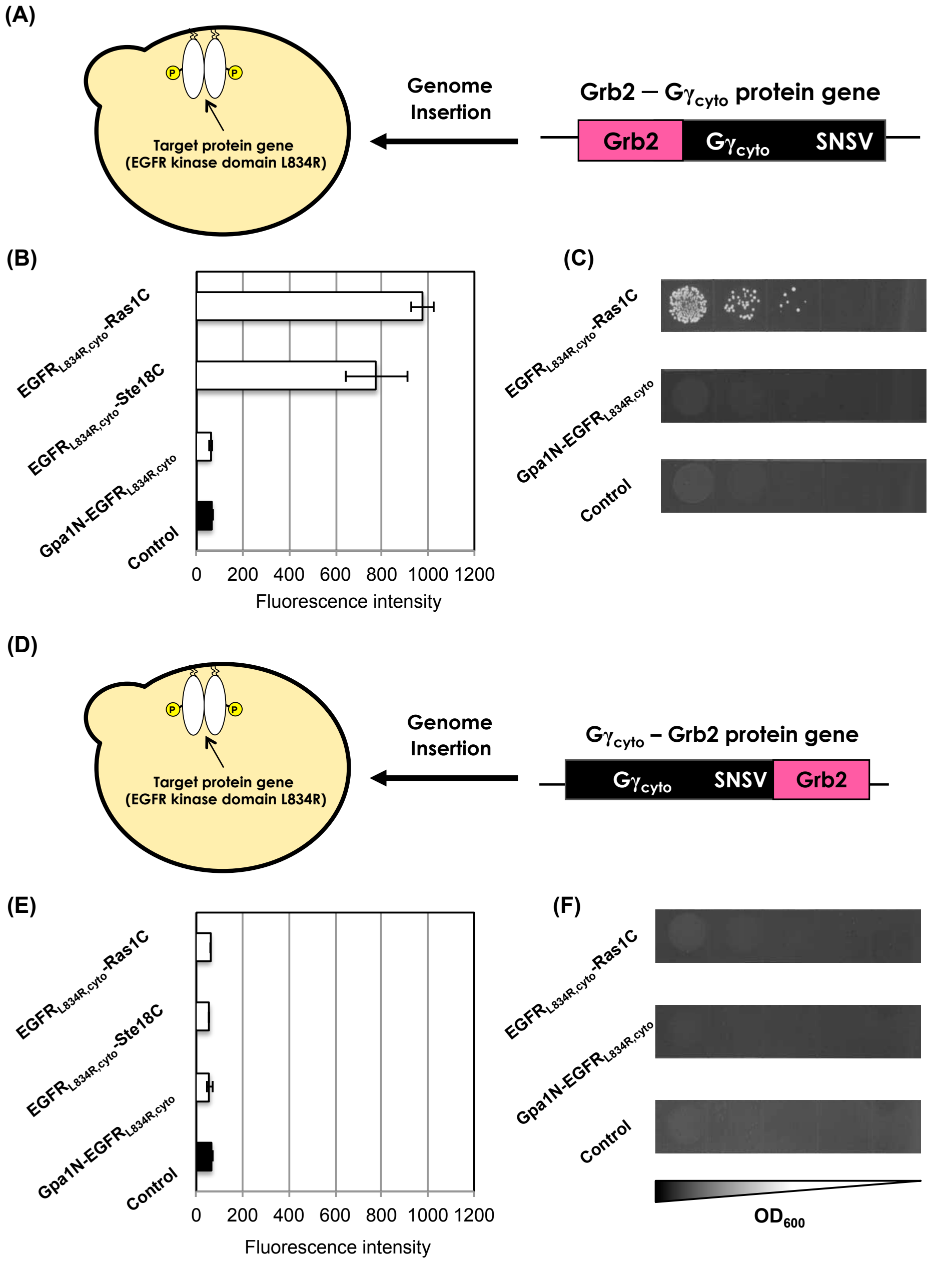

Figure S6. Examinations of the lipidation motifs for membrane-associated intracellular domain of EGFR and the fusion patterns between Gγ$_{cyto}$ and Grb2.

(A) The Gγ recruitment system for membrane-associated intracellular domain of EGFR L834R mutant (EGFR$_{L834R,cyto}$) and C-terminally Gγ$_{cyto}$-fused Grb2 (Grb2-Gγ$_{cyto}$). (B and C) The flow cytometric analyses and mating growth assay. Fluorescence and growth intensities of the engineered strains expressing N-terminally (Gpa1N) and C-terminally (Ras1C and Ste18C) membrane-associated EGFR$_{L834R,cyto}$ and cytosolic Grb2-Gγ$_{cyto}$ via stable integration into the yeast chromosome. The control yeast shows the strain without the expression of ‘Y$_1$’ fused to Gγ$_{cyto}$ (MC-ErC in Table 1). (D) The Gγ recruitment system for membrane-associated EGFR$_{L834R,cyto}$ and N-terminally Gγ$_{cyto}$-fused Grb2 (Gγ$_{cyto}$-Grb2). (E and F) The flow cytometric analyses and mating growth assay. Fluorescence and growth intensities of the engineered strains expressing N-terminally (Gpa1N) and C-terminally (Ras1C and Ste18C) membrane-associated EGFR$_{L834R,cyto}$ and cytosolic Gγ$_{cyto}$-Grb2 via stable integration into the yeast chromosome. The control yeast shows the strain without the expression of ‘Y$_1$’ fused to Gγ$_{cyto}$ (MC-ErC in Table 1).
